# The *g*-Strained EPR Line Shape of Transition-Ion Complexes and Metalloproteins: Four Decades of Misunderstanding and Its Consequences

**DOI:** 10.3390/molecules30153299

**Published:** 2025-08-06

**Authors:** Wilfred R. Hagen

**Affiliations:** Department of Biotechnology, Delft University of Technology, Building 58, Van der Maasweg 9, 2629 HZ Delft, The Netherlands; w.r.hagen@tudelft.nl

**Keywords:** EPR, *g*-strain, powder pattern, line shape, spin counting, non-colinearity, power saturation, iron–sulfur cluster, complex I, NADH dehydrogenase

## Abstract

Analysis of the EPR of dilute transition-ion complexes and metalloproteins in random phases, such as frozen solutions, powders, glasses, and gels, requires a model for the spectral ‘powder’ shape. Such a model comprises a description of the line shape and the linewidth of individual molecules as well as a notion of their physical origin. Spectral features sharpen up with decreasing temperature until the limit of constant linewidth of inhomogeneous broadening. At and below this temperature limit, each molecule has a linewidth that slightly differs from those of its congeners, and which is not related in a simple way to lifetime broadening. Choice of the model not only affects precise assignment of *g*-values, but also concentration determination (‘spin counting’), and therefore, calculation of stoichiometries in multi-center complexes. Forty years ago, the theoretically and experimentally well-founded statistical theory of *g*-strain was developed as a prime model for EPR powder patterns. In the intervening years until today, this model was universally ignored in favor of models that are incompatible with physical reality, resulting in many mistakes in EPR spectral interpretation. The purpose of this review is to outline the differences between the models, to reveal where analyses went astray, and thus to turn a very long standstill in EPR powder shape understanding into a new start towards proper methodology.

## 1. Introduction

Paramagnetic powders, glasses, gels, frozen solutions, solutions of macromolecules, and cryogenic condensates of gasses are all forms of matter in which the individual paramagnetic molecules are randomly oriented with respect to each other and with respect to the external magnetic-field vector. Their EPR spectra are said to exhibit a ‘powder pattern’, in which the orientation of the magnetic field with respect to a Cartesian axes system, defined at the paramagnetic center of a specific molecule, is determined by the three direction cosines that the field vector *B* makes with the axes *x*, *y*, and *z*. The resonance condition of an *S* = 1/2 system of orthorhombic symmetry is then defined as

**Figure 1:**
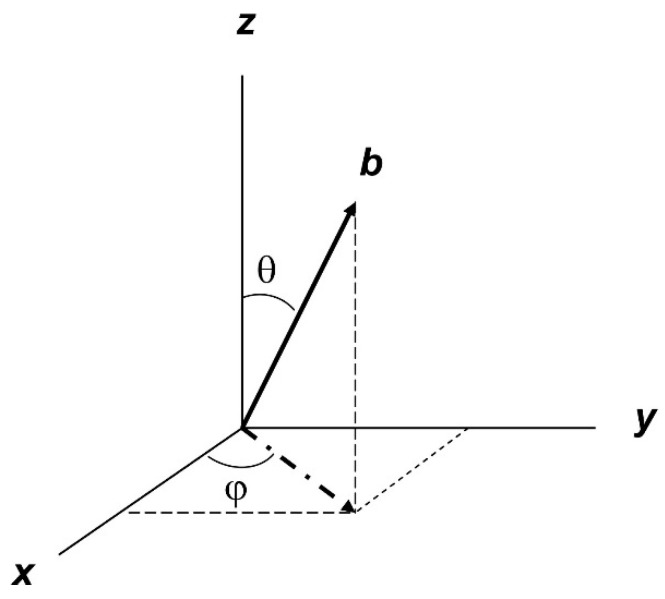
The orientation of a unit-length vector *b* along the field *B* in the Cartesian axes system *xyz* is defined by the polar angles *θ* between *b* and the *z*-axis and *φ* between the projection of *b* on the *xy* plane and the *x*-axis.

(1) hν=gβB

in which *h* is Planck’s constant, *ν* is the microwave frequency, *β* is the Bohr magneton, and

(2) $$g\left(cos\theta ,\phi \right)=\sqrt{L\cdot g^{2}\cdot L}=\sqrt{l_{x}^{2}g_{x}^{2}+l_{y}^{2}g_{y}^{2}+l_{z}^{2}g_{z}^{2}}$$

with the polar angles between *B* and *xyz* defined as in Figure 1.

Furthermore,

(3) $$L=\left(l_{x},l_{y},l_{z}\right)$$

(4) $$\overset{̿}{g}=\left(\begin{array}{ccc} g_{x} & 0 & 0\\ 0 & g_{y} & 0\\ 0 & 0 & g_{z} \end{array}\right)$$

(5) $$l_{x}=sin\theta cos\phi ;\;l_{y}=sin\theta sin\varphi ;\;l_{z}=cos\theta$$

**Figure 2:**
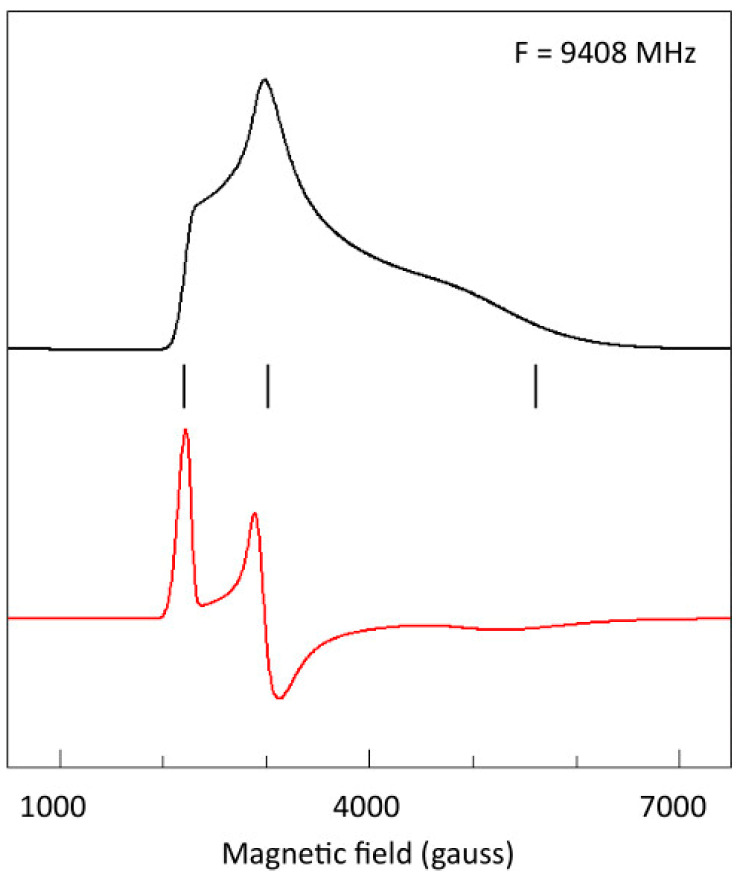
Example of an EPR absorption powder pattern (black trace) of rhombic symmetry, that is, with *g*_z_ ≠ *g*_y_ ≠ *g*_x_, affording a first derivative (red trace) with clear ‘spectral features’ (absorption, derivative, negative absorption) associated with the principal *g*-values. Note the pronounced variation in width over the three spectral features. The spectrum is from cytochrome *c* with *g*-values (indicated with vertical bars) 3.056, 2.228, 1.20 as determined by simulation based on *g*-strain [2].

The powder pattern is then defined by the occurrence of a large number (of the order of Avogadro’s number) of random combinations of (cos*θ*, *φ*) on a unit sphere comprising all possible orientations of *b* in Figure 1. The pattern can be numerically simulated with a much smaller number (of the order of 100 × 100) of (cos*θ*, *φ*) combinations with equidistant steps over the intervals 0 ≤ cos*θ* ≤ 1 and 0 ≤ *φ* ≤ π/2, that is, over one octant of the unit sphere. This step definition ensures division of the surface of the unit sphere into sub-surfaces of equal area. Several alternative step algorithms have been proposed to improve CPU efficiency ([1] and refs. quoted therein); however, since they all converge to the same end result at a high enough number of steps, choosing among them is irrelevant to the theme of this review. An example of a generated powder pattern is given in Figure 2.

The EPR linewidth in a first derivative powder pattern is operationally defined as the observed width of the three features of the pattern, that is the low-field absorption-shaped line, the intermediate-field derivative-shaped line, and the high-field negative absorption-shaped line.

**Figure 3:**
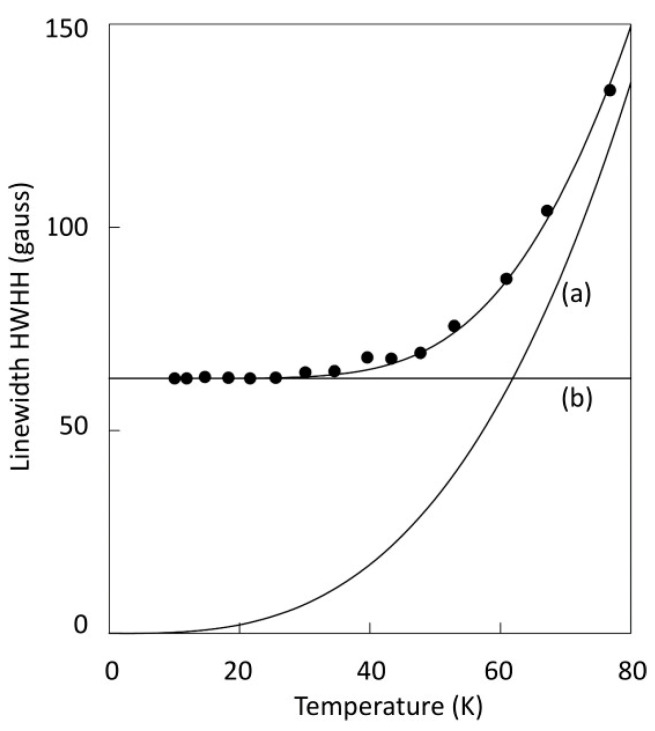
Linewidth of the *g*_z_ feature of cytochrome *a* in the enzyme cytochrome *c* oxidase as a function of temperature. The width can be deconvoluted into two components, one that sharpens with reducing temperature (**a**) and one that is independent of the temperature (**b**). The first one is associated with homogeneous broadening by spin-lattice relaxation characterized by a Lorentzian line shape. The second one is associated with inhomogeneous broadening by conformational distribution characterized by a Gaussian line shape. Data taken from [3].

When the spectrum of a dilute *S* = 1/2 system is recorded at a relatively high temperature, the shape of the EPR line is Lorentzian and its width is related to the spin-lattice relaxation rate of the spin system. Lowering the observation temperature affords a progressive sharpening-up of the line until at and below a given value for *T*, no more sharpening is found, and the shape has converted into a Gaussian line. An example is given in Figure 3.

**Figure 4:**
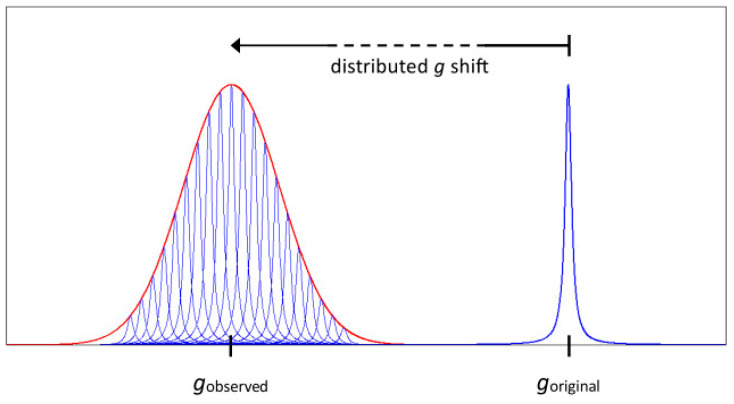
An EPR Lorentzian resonance line (blue) is distributed by a yet-to-be-defined cause, resulting in an inhomogeneously broadened Gaussian line (red). The original and the actual *g*-value may or may not coincide, depending on whether the cause of the *g*-shift is intrinsic or external to the electronic Zeeman interaction.

The high-temperature Lorentzian line is said to be homogeneously broadened, where the line represents a single spin packet, that is, a group of single-phased spins that all experience the same magnetic field. Contrarily, the low-temperature Gaussian line is taken to present inhomogeneous broadening, that is, comprising a collection of sharp Lorentzian spin packets, each subject to a different magnetic field, as illustrated in Figure 4.

Interpretation of the line as having a Gaussian shape corresponds to it being caused by a random distribution of spin-packet resonance fields. Experimental support is found in a very large number of EPR observations of at least very approximately Gaussian lines at X-band (ca 9–10 GHz) frequencies on dilute *S* = 1/2 systems, although a formal argument for its nature (including its presumed linearity in magnetic-field space) appears to have never been developed in the EPR literature. In the first derivative of a Gaussian line the peak-to-negative-peak field separation corresponds to two times the standard deviation, *σ*, of the random distribution. For a Gaussian absorption line, sigma is equal to 0.850 times the half width at half height. In EPR practice, definition of the width differs per author as either one sigma, two sigma, half width at half heigh (HWHH,) or full width at half height (FWHH).

In systems of lower than cubic symmetry, the value of *g* depends on molecular orientation with respect to the field vector (Equation (2)). Similarly, the line width in these systems can be anticipated to exhibit angular dependency, and knowledge of its precise form will be required in quantitative analysis by spectral simulation. We are therefore faced with the task of hypothesizing an angular dependence, preferably based on some theory, and then to test its validity by multiple observations.

A first attempt at this goal was made in 1965 by Johnston and Hecht [4], who assumed the null hypothesis that the linewidth’s angular dependence does not differ from the *g*-value’s angular dependence, that is, there is a width tensor, and the same coordinate transformation that diagonalizes the *g*-tensor also diagonalizes this width tensor. Thus, from Equation (2), rewritten as

(6) $$g^{2}\left(\theta ,\varphi \right)=l_{x}^{2}g_{x}^{2}+l_{y}^{2}g_{y}^{2}+l_{z}^{2}g_{z}^{2}$$

it was posed that

(7) $$w^{2}\left(\theta ,\varphi \right)=l_{x}^{2}w_{x}^{2}+l_{y}^{2}w_{y}^{2}+l_{z}^{2}w_{z}^{2}$$

with *w* in field units of gauss, that is, assuming a line shape linear in field space. No theoretical framework was given but note that Equation (7) implies some distribution in the *g*-value at any orientation.

When a spin system of orthorhombic symmetry is subject to interaction with a central (metal) nuclear spin, the first-order hyperfine splitting for a frequency-swept spectrum is

(8) $$A^{2}\left(\theta ,\varphi \right)=\left(l_{x}^{2}g_{x}^{2}A_{x}^{2}+l_{y}^{2}g_{y}^{2}A_{y}^{2}+l_{z}^{2}g_{z}^{2}A_{z}^{2}\right)/g^{2}(\theta ,\varphi )$$

In 1967, Venable [5] proposed that in a system subject to unresolved central hyperfine interaction, the linewidth anisotropy would, in analogy to Equation (8), be of the form

(9) $$w^{2}\left(\theta ,\varphi \right)=\left(l_{x}^{2}g_{x}^{2}w_{x}^{2}+l_{y}^{2}g_{y}^{2}w_{y}^{2}+l_{z}^{2}g_{z}^{2}w_{z}^{2}\right)/g^{2}(\theta ,\varphi )$$

Since, however, in practice, EPR spectra are rarely recorded as a frequency scan, we should take the field-scan analogy of the hyperfine interaction:

(10) $$A^{2}\left(\theta ,\varphi \right)=\left(l_{x}^{2}g_{x}^{4}A_{x}^{2}+l_{y}^{2}g_{y}^{4}A_{y}^{2}+l_{z}^{2}g_{z}^{4}A_{z}^{2}\right)/g^{4}(\theta ,\phi )$$

and the linewidth analogy on a field scale then becomes:

(11) $$w^{2}\left(\theta ,\varphi \right)=\left(l_{x}^{2}g_{x}^{4}w_{x}^{2}+l_{y}^{2}g_{y}^{4}w_{y}^{2}+l_{z}^{2}g_{z}^{4}w_{z}^{2}\right)/g^{4}(\theta ,\varphi )$$

Since *multiple* central hyperfine interactions cannot occur in mononuclear complexes, Equation (11) has been subsequently taken to describe the linewidth anisotropy due to unresolved superhyperfine splittings from ligand nuclei, for example, by Aasa et al. [6,7].

## 2. Linewidth Frequency Dependence

**Figure 5:**
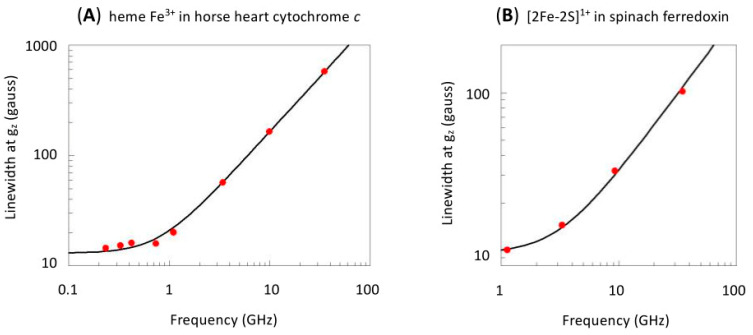
Linewidths in examples from two classes of metalloproteins. Widths are expressed in gauss and measured as FWHH at the *g*_z_ feature of the *S* = 1/2 spectrum. Data for the ferric heme spectrum of equine heart cytochrome *c* were combined from refs. [2,8,9]; data for the [2Fe-2S]^+^ spectrum of ferredoxin from spinacia oleracea are from ref. [10].

How can we experimentally test the validity of the above (and other) linewidth algorithms? The two models (Equation (7) versus Equation (11)) differ completely in their predicted dependence on the microwave frequency. Since the electronic Zeeman interaction is linear in the frequency, the field position of *g* is linear in the field (Equation (1)). Equally, a distribution in *g* should lead to a linewidth, expressed in field units, that increases linearly with the frequency. Contrarily, hyperfine interactions are independent of the external field, and a linewidth due to unresolved (super) hyperfine interactions, and expressed in field units, should be independent of the employed frequency. Published data are sparse; in several papers, I reported numbers for the low-spin ferric heme protein cytochrome *c* and for the ferredoxin from spinach leaves, which are compiled in Figure 5.

The graph for the low-field peak of the hemoprotein cytochrome *c* is flat at very low frequencies, presumably due to a combination of broadening by many unresolved superhyperfine splittings and broadening by intermolecular dipole–dipole interaction [2]. However, from ca 1 GHz onwards, the width on a field scale is linear with the frequency and thus dominated by a *g*-value distribution. The plot for the ferredoxin is essentially linear from the X-band onwards, but it levels off versus lower frequencies, most probably attesting to the presence of unresolved superhyperfine interaction [10]. The bottom line is that specifically in the X-band, the frequency at which most data are taken for accurate numerical simulation, the low-temperature spectra of these two classes of compounds are likely to be dominated by inhomogeneous broadening from *g*-distributions.

## 3. Linewidth (Non-)Colinearity

**Figure 6:**
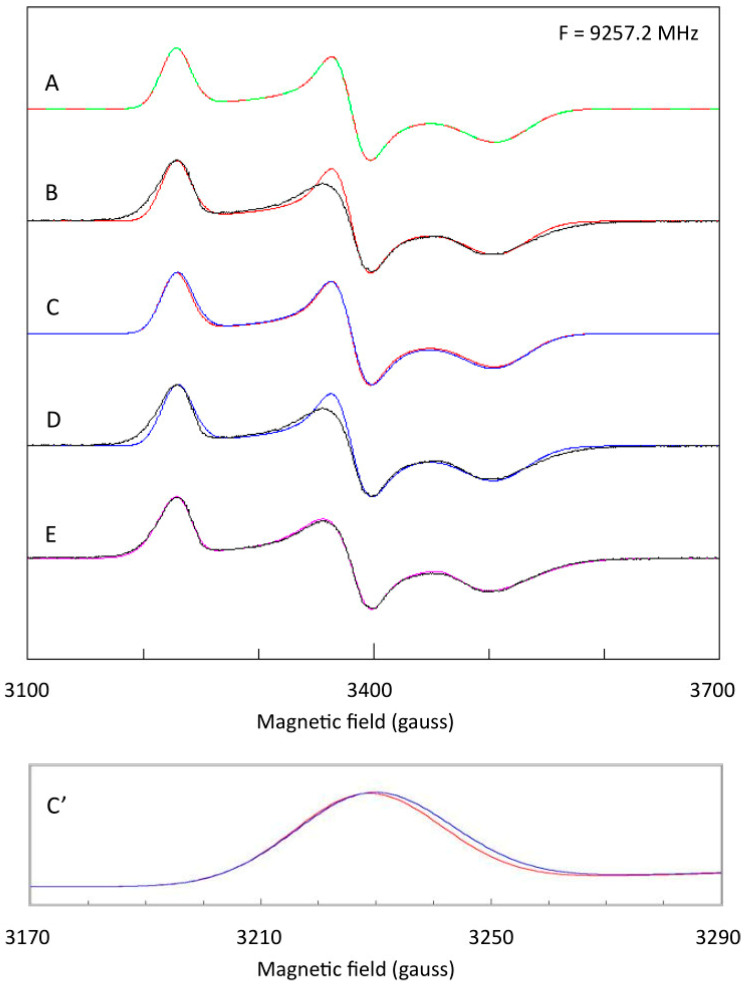
EPR spectrum and simulations of spinach ferredoxin [2Fe-2S]^1+^ cluster. The experimental spectrum is in black in traces B, D, and E. The green simulation in trace A is based on Equation (7). The red simulation in traces A, B, and C is based on Equation (11). For the blue simulation in traces C, D, the linewidth of the red simulation (in gauss) was converted to colinear *g*-strain; then, the simulation was calculated in *g*-space and converted to *B*-space. The magenta simulation in trace E is based on Equation (17) of the non-colinear *g*-strain. Trace C’ is a blow-up to show the effect of conversion of *g*-space to *B*-space (blue) compared to a ‘classical’ simulation in *B*-space. Simulation parameters (green, red): *g* = 1.8848, 1.9561, 2.0479; *w* = 31.8, 15.2, 15.2 gauss; (blue) ∆ = 0.0171, 0.0088, 0.0097; (magenta) *g* = 1.881, 1.956, 2.0502, ∆ = 0.021, 0.011, 0.0107, ∆_xy, xz, yz_ = 0.0115, −0.0150, −0.0030. The experimental spectrum was taken from ref. [10].

Of note, the two broadening mechanisms (Equation (7) and Equation (11)) cannot be distinguished based on spectral shape. Although they differ in angular variations, their linewidth tensors are both colinear with the *g*-tensor, and since the powder patterns are dominated by the canonical features, that is, the features for which the magnetic-field vector is parallel to one of the molecular axes, they render simulated powder pattens that are essentially identical (see Figure 6A). When we use either of them to simulate the experimental spectrum of spinach leaf ferredoxin, they are found to do a poor job, as they do not reproduce the tailing of the *g*_z_ absorption feature towards low field, nor the tailing of the *g*_x_ negative absorption feature towards high field, nor the asymmetry in the *g*_y_ derivative feature (Figure 6B).

Both algorithms assume Gaussian distributions that are linear and symmetric on a magnetic-field scale. For the *g*-value distribution in Equation (6), this assumption is rather unlikely to be true. A Gaussian distribution in *g*-values should be linear and symmetric on a *g*-value scale and, therefore, should be skewed on a field scale (=1/*g* scale). If we calculate the *g*-distributed spectrum in *g*-space followed by transformation to *B*-space [8], then the two approaches do differ moderately (Figure 6C). Note also that the apparent average *g*-values read out directly from the field-swept spectrum now deviate from the real *g*-values. Remarkably, except in my own work [8,10,11], and subsequently in that of John Pilbrow [12], this obvious notion has been generally ignored in the literature, up to this day. Since the difference between the two is not pronounced, we would expect also the simulation with Gaussians symmetrical on a *g*-value scale to fall short of being able to faithfully reproduce the experimental ferredoxin spectrum, which is indeed borne out by the fit in Figure 6D.

Clearly, real powder patterns are often more complex than predicted by *g*-distributed linewidth tensors colinear with the *g*-tensor (Figure 6D). This debunking of the two models (Equations (7) and (11)), above, calls for an alternative model. In 1981, working towards my Ph.D. in the lab of Siem Albracht in Amsterdam, I proposed the *g*-distribution to be induced by an external stress (mechanical and/or electrical in origin), resulting in the *g*-value to be shifted via a linear coupling to a local strain tensor whose elements were normally distributed [8,13]. Strain adds an extra term to the spin Hamiltonian for *S* = 1/2,

(12) $$H=\sum \varepsilon_{ij}T_{ijkl}B_{k}S_{l}$$

in which ***T*** is a fourth-rank tensor coupling the symmetrical strain tensor ***ε*** to the Zeeman field. My starting point was the spin Hamiltonian for an *S* = 1/2 system subject to strain due to [14]:

(13) $$g_{eff}=g+\left(R_{1}/\beta \right)\left(\varepsilon_{xx}+\varepsilon_{yy}+\varepsilon_{zz}\right)\;+\left(2R_{2}/3\beta \right)\left\{\varepsilon_{xx}\left(3l_{x}^{2}-1\right)+\varepsilon_{yy}\left(l_{y}^{2}-1\right)+\varepsilon_{zz}\left(3l_{z}^{2}-1\right)\right\}\;+\left(2R_{3}/\beta \right)\left(\varepsilon_{xy}l_{x}l_{y}+\varepsilon_{xz}l_{x}l_{z}+\varepsilon_{yz}l_{y}l_{z}\right)$$

in which the *ε*_ij_-values are the elements of a symmetric strain tensor and the *R*-values are the three coupling coefficients to which the coupling tensor ***T*** in Equation (12) can be reduced in cubic symmetry. Then, taking the elements of the strain tensor to be normally distributed with a full width at half height equal to ∆*ε*_ij_, Equation (13) can be rewritten in terms of a linewidth in energy units [8].

(14) $$w_{g\;strain}(\theta ,\varphi )=\left|{∆}_{xx}l_{x}^{2}+{∆}_{yy}l_{y}^{2}+{∆}_{zz}l_{z}^{2}+2{∆}_{xy}l_{x}l_{y}+2{∆}_{xz}l_{x}l_{z}+2{∆}_{yz}l_{y}l_{z}\right|$$

or in matrix notation

(15) $$w_{g\;strain}(\theta ,\varphi )=|L\cdot ∆\cdot L|$$

(16) $$∆=\left(\begin{array}{ccc} {∆}_{xx} & {∆}_{xy} & {∆}_{xz}\\ {∆}_{xy} & {∆}_{yy} & {∆}_{yz}\\ {∆}_{xz} & {∆}_{yz} & {∆}_{zz} \end{array}\right)$$

in which Equations (14) and (15) are now periodic over half the unit sphere. With the null hypothesis that restricting the coupling tensor ***T*** to cubic symmetry would still reproduce the salient features of an inhomogeneous linewidth determined by the *g*-strain distribution, the model was put to the test for its ability to faithfully reproduce real powder patterns. Indeed, simulations of spectra from low-spin hemoproteins [8] or from ferredoxins [10] were near perfect and certainly a big step forward compared to the limited ability of the conventional models of Equation (7) or Equation (11) to reproduce experimental data. The excellent correspondence between experimental spectra and simulations (cf [8,10]) suggested that it would not be meaningful to allow for a symmetry-unrestricted fourth-rank coupling tensor ***T*** with formally up to 81 independent fitting parameters.

With the apparent success of the approach formalized in Equation (15), still a nagging feeling remained regarding the questions of why the successfulness, and what would be its physico-chemical origin. I therefore moved in 1982 to the biophysics research group of Dick Dunham and Dick Sands at the University of Michigan in Ann Arbor, where physical chemists David Hearshen, Dick Dunham and I, with support from numerical analyst Len Harding and physicist Dick Sands, spent a number of man years in a quest to develop a formal statistical theory of *g*-strain [11,15,16]. We made the central assumption that *g* is a random variable resulting from a distribution in the Zeeman parameters that finds its origin outside the *g*-value itself. We then wished to determine the expectation value and the variance of *g*. This led to a heap of mathematics, which I will not reproduce here because it has been comprehensively documented in [15]. The key result, in case of full (positive or negative) correlation between the *g*-value itself and the random variables that cause *g*-strain, was a broadening of the inhomogeneous EPR line width:

(17) $$w_{g\;strain}\left(\theta ,\varphi \right)=\left|\Lambda \cdot \Delta \cdot \Lambda \right|/g(\theta ,\varphi )$$

in which

(18) $$\Lambda =\left(l_{x}^{2}g_{x},l_{y}^{2}g_{y},l_{z}^{2}g_{z}\right)$$

Note the close correspondence between Equations (15) and (17), the only difference being in the vector *L* that defines the orientation of the magnetic field in the *g*-tensor axes system, which in the statistical theory becomes a *g*-weighted vector with *g*-normalization. This does change the angular dependence of the linewidth at, and in between, the canonical orientations, so Equation (15) is not a valid substitute for Equation (17). Figure 6E gives a simulation, based on Equation (17), of the spinach ferredoxin spectrum. In terms of quality of fit, there is not much left to wish for. A recent version of my *g*-strain simulator (executable and full source listings) was provided in the freely available Supporting Materials to ref. [2].

One should bear in mind that, as with Equation (15) (symmetry-restricted coupling tensor ***T***), Equation (17) is also a special case of a general solution, namely, that of full correlation. If the random variables are not fully correlated, that is, if the correlation coefficients are not equal to ± unity, then they become variables to be fitted, and the general form of Equation (17) becomes five-dimensional instead of three-dimensional [15]. Experimental exploration of this possibility suggests that loosening the condition of full correlation has only minor effects on the powder pattern [16].

All in all, it had become abundantly clear that EPR spectra reflect metalloproteins subject to conformational distributions at their metal sites, and presumably also elsewhere in the proteins. A question that remains difficult to answer in a quantitative manner is to what extent this distribution is intrinsic to the nature of proteins, and/or to what extent it is induced by external influences. Crystal structures of proteins generally show atomic displacement parameters attesting to flexibility and movement. On the other hand, the *g*-strained EPR powder pattern is influenceable by an experimentalist with external means. High pressure increases *g*-strain [10]. Addition of cosolvents (e.g., glycerol, methanol) results, upon freezing, in decreased *g*-strain (increased spectral resolution), which suggests that limitation of ice crystal growth reduces external stress. In a similar vein, Simon de Vries developed a rapid-freeze instrument with a record dead time of 80 μs [17,18]. Shortly before he unexpectedly passed away, he showed me EPR spectra of Cu(II) ions in TRIS buffer, which exhibit superhyperfine structure from four nitrogen ligands. When the complex was frozen in ca 100 μs on a rotating cryogenic cold plate, the SHF structure was quite significantly better resolved than in complex immersed either in liquid nitrogen or isopentane, suggesting less developed *g*-strain by limitation of ice crystal growth in time. Whereas more elaborate considerations may be developed for specific clusters of transition-ion complexes (cf, e.g., [16]), a possibly generally useful picture may be to visualize a metalloprotein in frozen aqueous solution, as subject to an external hydrostatic stress from ice microcrystals as the single random variable, which is translated by the specific 3D structure between the surface of the protein and the metal site into a distributed deformation described by a linewidth tensor that is non-colinear with the average *g*-tensor. From a practical perspective, a perhaps more pressing question is how we should deal with the implications of the occurrence of *g*-strain in quantitative spectral analysis.

## 4. Key Consequences of *g*-Strain

**Figure 7:**
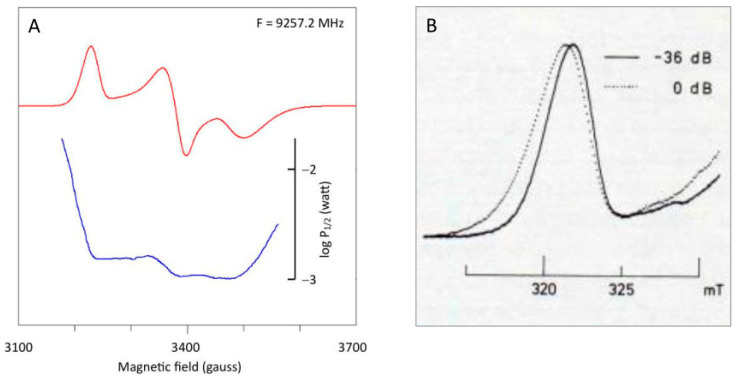
Differential saturation over the powder pattern of spinach ferredoxin. (**A**) the microwave power level required for half saturation was determined at every point of the digital spectrum. Note the particularly pronounced decrease in saturation into the extreme wings of the spectrum. Data taken from ref. [10]. (**B**) The *g*_z_ peak was taken at non-saturating versus saturating power at a temperature of 24 K, a frequency of 9236 MHz, and with a low modulation frequency of 1 kHz to avoid deformation (partial integration) by passage effects (reprinted from [13]).
  Clearly, attempts to saturate the low− and high-field peaks will lead to strong deformation of shape with apparent increase in intensity towards extreme fields. In other words, differential saturation is inherent in *g*-strain.
    Note, in passing, that in the iron–sulfur protein literature (specifically in the literature on Complex I, which is the subject of the next sections), a variant of Equation (19) has been used
  (20) $$S\propto \frac{\sqrt{P}}{{\left(1+P/P_{1/2}\right)}^{b/2}}$$
  in which *b* is an ‘inhomogeneity parameter’ that is usually taken to run from *b* = 1 (inhomogeneous broadening) to *b* = 2 (homogeneous broadening). A justification for this form was never given. For example, Equation (20) was used in refs. [19,20,21] with reference to previous papers [22,23] that simply pose the equation without explanation or reference to any original source. The equation originates from a 1953 paper by Portis, who, however, deduced it for—long extinct—spectrometers with a modulated microwave source [24]. Its use on data from field-modulated spectrometers has no theoretical basis but is supported by the good fits it gives to experimental data. Thus, the parameter *P*_1/2_ effectively characterizes saturation, hopefully, in a semi-quantitative manner. Furthermore, I have made the point that for lines that are inhomogeneously broadened by *g*-strain, an adjustable inhomogeneity parameter, *b*, has no meaning beyond that of a fudge factor [10].
(5) Methods have been described in the literature to determine spin concentration on the basis of the first integral of one of the peaks in the powder pattern [6]. If these peaks are asymmetric due to non-colinear *g*-strain, then the method may lead to an underestimation of the spin concentration of the order of some 25% [8]. In cases in which the peak is poorly separated from the derivative feature, quantifications have been based on the surface under one-half of the peak (the half on the extreme-field side) [25]. For *g*-strained systems, this may well lead to an additional error in spin count.

The occurrence of *g*-strain in EPR powder patterns implicates the following manifestations:

(1) The inhomogeneous line shape is a normal distribution in *g*-space; in *B*-space, the line shape is slightly skewed, and the peak does not correspond exactly with the average *g*-value (cf Figure 6C’).
(2) The three main features in the powder pattern are generally asymmetric: the low-field absorption-shaped peak skews towards low field; the lobes of the derivative-shaped peak have unequal amplitudes and different widths; the high-field absorption-shaped negative peak skews towards high field (cf Figure 6E).
(3) The possibility of full negative correlation implies that the linewidth may change sign (that is, go through zero) for a particular intermediate (*θ*, *φ*) orientation of the field vector in the molecular axes system; as a consequence, the number of orientations required to numerically generate a smooth powder pattern with no microcrystallinity artifacts may increase by a few orders of magnitude.
(4) Microwave power saturation over the powder pattern is not a constant. In particular, the low-field wing of the absorption peak and the high-field wing of the negative absorption peak are increasingly difficult to saturate towards extreme field, as illustrated for spinach ferredoxin in Figure 7, in which half-saturation values were determined with Equation (19).
  (19) $$S\propto \sqrt{\frac{P}{1+P/P_{1/2}}}$$
  in which *S* is the signal amplitude, *P* is the microwave power, and *P*_1/2_ is the power for which the signal is half-saturated.

## 5. Forty Years of Misinterpretations of the Key Consequences of *g*-Strain

Clearly, if, for a group of compounds, such as low-spin hemes or low-spin iron–sulfur clusters, the EPR is generally conditioned by *g*-strain, the above key points should be taken into account in line-shape simulation, spin counting, stoichiometry determination, deconvolution by differential saturation, etc., especially in the analysis of multi-center molecules. Failure to take these boundary conditions into account is very likely to lead to significant errors of interpretation (as shown in the second next section). Remarkably, over the last four decades, the above key points have almost universally been ignored. Two typical approaches can be identified.

The first approach, which has dominated the first two decades following the development of a rigorous *g*-strain theory, was to altogether ignore the literature on *g*-strain, as well as the literature on multi-frequency EPR that showed linearity of linewidth with frequency, and to simply simulate spectra on a field scale with an improper equation (e.g, Equation (11)) for the angular variation of the line width (out of many publications; see, for example, the iron–sulfur papers [26,27,28,29,30,31,32,33,34,35]). These studies are in conflict with the *g*-strain manifestations 1–3, above. Possibly even more regrettable (see second next section) would be ignoring the validity of manifestation 4, which states that deconvolution of multi-component spectra with differential saturation is generally an invalid method for *g*-strained systems. This mistake, which may have initially been made out of ignorance, more recently has been specifically promoted as a defendable approach. Troy Stich who recently (2021) wrote a chapter on characterization of iron–sulfur clusters using EPR in a book dedicated to methods and protocols for Fe-S cluster analysis [36] claims: ‘According to Hagen, it is worth “pointing out that the theory of *g*-strain implies continuously varying saturation behavior over the powder envelope of a single species; therefore, differential saturation is not a reliable criterion for deconvolution of these complex spectra” [37]. While this is technically correct, as a practical matter, differential saturation behavior *can* (my italicization) be used to indicate which EPR features in a spectrum of a complex mixture of paramagnetic species arise from a single species’. My reading of this statement: although *g*-strain is a reality (technically correct), let us assume (as a practical matter) it does not exist. Overtly or silently adopted, this approach has contaminated four decades of literature, as amply illustrated in the second next section.

The second approach, dominating the last two decades until the present time, was to claim that *g*-strain broadening had, in fact, been incorporated in spectral analyses, be it only in the form of a linewidth tensor colinear with the *g*-tensor (that is, assuming ∆_xy_, ∆_xz_, and ∆_yz_ in Equation (17) to be identically equal to zero) and with a Gaussian line shape that is linear on a magnetic-field scale (out of many papers, see, for example, [20,38,39,40,41,42,43,44,45,46,47]). No arguments were ever provided for general colinearity of the Zeeman interaction and *g*-strain nor for the magical linearity in *B*-space. My guess would be that the only ‘justification’ is to be found in the fact that the software used in all these studies [48,49] simply does not provide any possibility to include non-colinear *g*-strain in *g*-space.

In the next two sections, I will illustrate, in the case of the respiratory enzyme Complex I, or proton-pumping NADH:quinone oxidoreductase, that these two approaches have resulted in authors making the same mistakes over and over again with the net result that zero progress has been made over the last four decades in quantitative EPR spectral-shape analysis of complex iron–sulfur cluster systems.

## 6. Complex-I: Stoichiometry of Signals N1b-N4

The first EPR recording of bovine Complex I (45 different subunits) occurred as early as 1960, and thus more or less coincided with the discovery of what we now know as iron–sulfur clusters [50]. This was followed in the early 1970s by a number of attempts by several groups to identify signals from different centers [51,52,53,54,55,56,57], culminating in a comprehensive attempt to deconvolute the complex spectrum into single spectral (and thus single molecular structural) components by Tomoko Ohnishi in 1975 [58] in an EPR study of pigeon heart mitochondria and submitochondrial particles, that is, systems containing all respiratory complexes including the FeS enzymes Complex II (succinate dehydrogenase) and Complex III (coenzyme Q:cytochrome c oxidoreductase). What appeared to be a set of five separate EPR signals from iron–sulfur clusters was given the labels N1a, N1b, N2, N3, and N4 (with N for NADH dehydrogenase) [58]. N1a was only observed at low redox potential of ca −0.4 V (the enzyme’s substrate NADH has E_m,7_ = −0.32 V). I defer consideration of this cluster to the next section. Also, two more sets of signals labeled N5 and N6, which were recorded under heavily saturating conditions (temperature close to 4.2 K; 20 mW microwave power), will be evaluated in the next section. N3 and N4 were reported to be spectroscopically almost indistinguishable with *g*_x_ = 1.87–1.88; *g*_y_ undetermined; and *g*_z_ = 2.10–2.11, so their separate labeling remained inconclusive. Two years later (1977), Siem Albracht undertook a re-analysis and also made the very first attempt at establishing the signals’ stoichiometry (that is, the spin concentration represented by each individual spectrum in relation to the chemical concentration of the enzyme) in his study of the purified bovine heart Complex I [59]. With Albracht adopting the signal labeling of Ohnishi (N1a, N1b, N2, N3, N4), a controversy arose which has endured till this day (e.g., [33,60]). Ohnishi’s N1a was not detectable under the EPR conditions used by Albracht; however, since he thought to perceive a small inhomogeneity in the signal of center N1b, he differentiated it into N1a and N1b. In a later joint study by the groups of Dunham, Ohnishi, and Albracht [61], it was concluded that the differentiation has no physical basis; therefore, in what follows, I will ignore it and use the label N1b for what Albracht originally called N1a plus N1b. A second problem of ambiguous labeling was created when Albracht found significantly different spectra for N3 (*g*_x_ = 1.884; *g*_z_ = 2.103) versus N4 (*g*_x_ = 1.863; *g*_z_ = 2.037), making it clear that Ohnishi had missed the much lower *g*_z_ value of N4. Albracht’s labeling was adopted by myself in 1985 [11] and by Kowal et al. in 1986 [62], but in the more recent literature, in particular, from Ohnishi’s group [63,64] and from the Hirst group [20,33,34,60,65,66], this labeling has been consistently interconverted to the original N3, N4 labeling by Ohnishi [58], although the latter was ambiguous due to low resolution and a missed *g*_z_-value.

With crystallographic and electron microscopic structural data becoming available in later years, combined with isolates from different species plus substructures and mutated structures, this numbering/labeling has undergone several rearrangements and extensions over time. The re-numbering bears relevance to the spatial location and the electron-transfer order of the clusters in the protein 3D structure as well as their association with different subunits; however, the actual labels are immaterial to the problem addressed here, which is the methodology associated with spin-count stoichiometry of bona fide single species EPR signals each with a well-defined *g*-tensor and linewidth tensor.

Although different methodologies result in different stoichiometries, the reported *g*-values are relatively insensitive to the approach taken, as can be seen in Table 1.

The different centers can thus be readily recognized in the spectrum by the following characteristics: N1b, with *g*_z_ = 2.02, is the only signal detected at *T* = 40 K; at lower *T* ≈ 12–17 K, N2 has *g*_z_ = 2.05; N3 has *g*_z_ = 2.10 and *g*_x_ = 1.88; N4 has *g*_x_ = 1.86.

The stoichiometry of FeS signals in Complex I has been expressed in the literature in absolute or in relative terms. The spin concentration has been absolutely related to the enzyme’s concentration or to the concentration of flavin (FMN) associated with the enzyme. The former has the disadvantage of experimental inaccuracy in the determination of a protein with an approximate mass of 950 kDa; flavin-concentration determinations are typically more accurate, but here, interference may arise due to loss of non-covalently-bound FMN from the enzyme. Relatively, the individual signals can be added in a certain ratio to end up in an overall sum spectrum that should come as close as possible to the enzyme’s EPR spectrum under defined conditions (here: NADH-reduced enzyme, and EPR not subject to microwave power saturation). The ratios can be expressed as stoichiometries by defining the concentration of one of the centers to be exactly equal to unity. They can also be expressed as percentage where the sum-intensity should be normalized to 100%. All four methods have been used; all are interconvertible when chemical concentration data are available.

**Figure 8:**
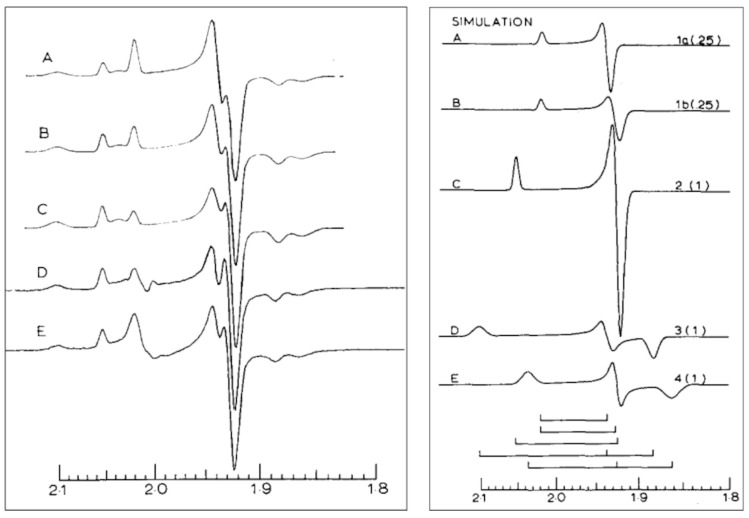
Albracht’s sum-simulations based on broadening by unresolved hyperfine interactions (**left**). Traces A–C are simulations in which the ratio of N1b (N1a + N1b in his notation) to N2-N3-N4 changes from 2:1 to 1:1 to 0.5:1, and they are compared to experimental data (trace D) from Complex I reduced with sodium dithionite + NADH (measured at 17 K), minus the spectrum of Complex I reduced with dithionite only (measured at 50 K), and to data (trace E) of enzyme reduced only with NADH (17 K). The (**right**)-hand panel gives the simulated spectra of the individual components with the indicated relative spin counts [59]. Note that N1a and N1b were later shown to be a single entity, which is now known as center N1b [61]. The used *g*-values are in the first entry of Table 1 for each center. Reprinted from [59] with permission from Elsevier.

Albracht’s 1977 analysis [59] was based on numerical simulation of the individual spectra, in which he used Equation (11) to model the broadening as due to unresolved ligand hyperfine splittings linear on a magnetic-field scale. He concluded that his best fit to the overall spectrum was obtained with a stoichiometry of 0.5:1:1:1 for N1b:N2:N3:N4 (Figure 8).

With reference to the concentration of FMN, the individually integrated spectra gave 0.40:0.78:0.56:0.86 for N1b:N2:N3:N4. Albracht’s conclusion was that three out of four clusters were approximately stoichiometrically present, while cluster N1b was present in approximately half the enzyme’s concentration. In subsequent years, he tried to find a biochemical interpretation for this non-integer stoichiometry in an asymmetric dimer model, in which one of the complex-I monomers did not contain N1b. The dimeric complex would contain different routes for the oxidation of NADPH versus NADH ([67] and refs. quoted therein; see also [68] for a model revision). Other groups typically found higher spin counts for N1b [11,33,60,62,65,69,70]. The asymmetric-dimer model (specifically: a possible 0.5 stoichiometry of N1b) has not been substantially discussed in the subsequent literature, and, in fact, appears to have eventually disappeared into oblivion.

**Figure 9:**
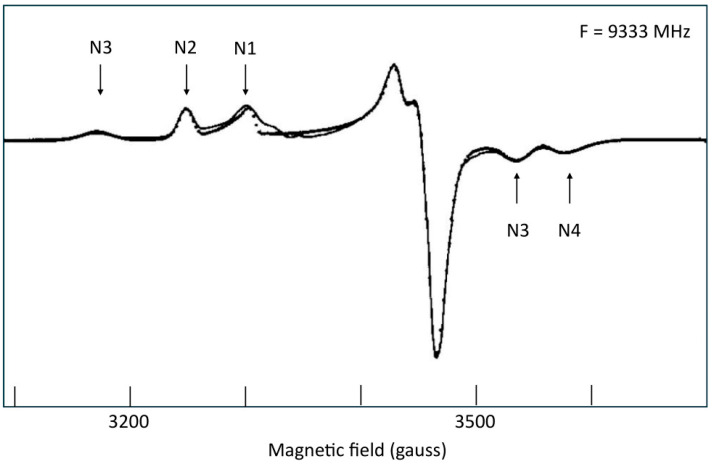
My sum-simulation based on fully correlated non-colinear *g*-strain. NADH-reduced Complex I (15 K; 0.2 mW) was simulated as a sum of N1b:N2:N3:N4 = 1:1:1:1, with the spectrum of each individual cluster defined by *g*-values (second entry of Table 1 for each center) and by a linewidth tensor containing six independent elements [11].

Upon completion of the statistical theory of *g*-strain [15], I took Albracht’s spectrum of NADH-reduced Complex I as a test case for the relevance of non-colinear *g*-strain to discriminate between enzymological interpretations [11]. I simulated the spectrum with four components, N1b, N2, N3, N4 in an ideal 1:1:1:1 ratio, and this generated a sum spectrum that almost perfectly fitted the experimental spectrum (Figure 9), and which was certainly a better fit than Albracht’s 0.5:1:1:1 one (Figure 8 left trace C versus E).

**Figure 10:**
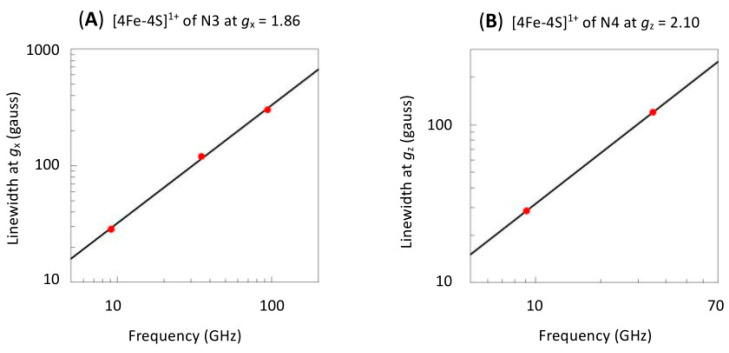
Linewidths of centers N3 and N4 are essentially proportional to the microwave frequency at and above X-band frequencies. Measurements were made as indicated on top of the figures. The proportional fits include the theoretical origin, which was set as point (0.01, 0.01) for the logarithmic scales. Data were taken from refs. [20,56,59].

From a numerical-analytical point of view, one can object that a better simulation is expected since the number of fitting parameters has been increased with three non-diagonal *g*-strain terms (∆_xy_, ∆_xz_, ∆_yz_) for each cluster, but the key justification here is the fact that, if *g*-strain occurs, then it is mandatory to explore its non-colinearity with the *g*-tensor. That the spectrum is dominated by *g*-strain is suggested from a comparison of the Q-band (35 GHz) [56] and the W-band (94 GHz) [20] spectra to the X-band spectrum showing strong similarity in *g*-space. A semi-quantitative underpinning of this conclusion is seen in Figure 10, in which linewidths, which I estimated from published-figure blowups, for the two extreme, almost isolated peaks of the spectrum, appear to be nearly proportional to the microwave frequency.

My naïve expectation at the time (1985) was not only that the 0.5 stoichiometry problem of N1b in Complex I would be considered resolved, but moreover that the statistical *g*-strain theory would from then on be generally embraced as the proper methodology for the analysis of multi-component EPR powder patterns. As indicated in the previous section, this wishful thinking did not materialize at all. What followed was two decades of completely silent denial, and then two decades up to this date of general adaptation of a simplified and deformed modification of the *g*-strain theory (namely, shapes symmetric in *B*-space and ∆ tensors always colinear with *g*-tensors). Here are some specific examples.

In 1995, the combined groups of Tomoko Ohnishi and Thorsten Friedrich reported on NADH-reduced Complex I from *Escherichia coli*. They analyzed a spectrum taken at 13 K and 10 mW microwave power using simulations based on symmetric broadening in *B*-space, presumably using Equation (7) or Equation (11), although no algorithm was specified, neither in the paper nor in its references [69]. They found the signal of a ‘new’ center, which they labeled N1c, and which was apparently only present in *E. coli* Complex I. Friedrich’s group later made the case that N1c is actually probably N1a, a center that is apparently not reducible by NADH in Complex I from other species [71]. The overall analysis afforded a stoichiometry of N1b:N1c:N2:N3:N4 = 0.52:0.06:1.00:1.32:0.75, with the caveat that N1b and N1c were ‘highly saturated’ under the employed conditions. The saturation characteristics of the other centers were not provided. One wonders how a center (N1b) can still contribute 50% of maximal intensity when it is ‘highly saturated’, and moreso, why one would attempt a stoichiometry determination under conditions of generally undetermined saturation characteristics. The authors did not comment on the non-integer stoichiometries found for the other centers N2, N3, N4, nor did they compare their results to the non-integer stoichiometry reported by Albracht [59] or the full-unit stoichiometry reported by me [11] for the bovine enzyme.

The group of Ohnishi, in collaboration with Weiss’s group had previously undertaken a similar analysis of Complex I in *Neurospora crassa*, but the report was ambiguous. The text claimed that EPR analysis of N1a:N2:N3:N4 stoichiometry afforded the numbers 0.7:1.0:1.0:1.0; however, the legend to the simulation claimed that all signals were added in a 1:1 ratio. Furthermore, the simulation was not overlaid over the experimental spectrum, and close inspection shows that some peaks in the simulation (*g*_z_ of N2 and *g*_z_ of N1) were of greater amplitude than in the experimental spectrum, while other peaks (*g*_x_ of N3 and *g*_x_ of N4) were actually smaller. The authors called this a ‘reasonably good fit’ [72].

A similar study (1995) of Ohnishi’s group in collaboration with Grohmann’s group on Complex I for potato tuber resulted in N1a:N2:N3:N4 ratios of 1.0:0.7:0.9:1.0. The quality of the fit to a rather noisy spectrum of small amplitude is difficult to evaluate [73].

**Figure 11:**
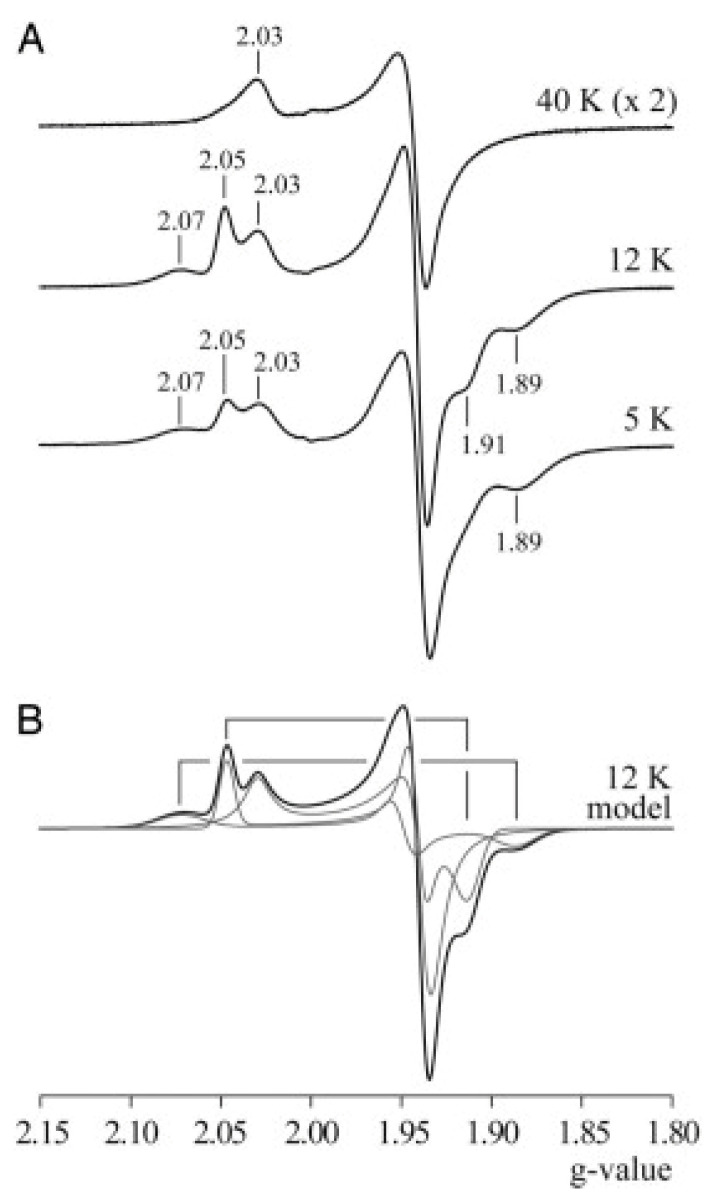
EPR spectra at different temperatures (**A**) and simulations (**B**) of dithionite-reduced subunit NuoG from Complex I of *E. coli* from Hirst’s group. The signal from center N1b recorded at 40 K ((**A**), top trace) is seen to be skewing towards extreme field. This observation was taken to imply that the individual line shape is Lorentzian. Subsequently, it was assumed (incorrectly) that this shape would not change (notably: sharpen) when lowering the observation temperature to 12 K, and the Lorentzian at 40 K was used to fit the 12 K spectrum (**B**). Reprinted from [33]; Copyright National Academy of Sciences.

In 2007, the group of Judy Hirst [33], studying subunit NuoG of *E. coli* Complex I, found that the spectrum of N1b at 40 K (when all other signals are broadened away) was asymmetrically tailing at *g*_z_ towards low field and at *g*_⊥_ towards high field (as one would expect in a non-colinearly *g*-strained spectrum) (Figure 11).

They decided that this must be due to the line shape being Lorentzian in *B*-space, and so they used a simulation of the 40 K spectrum in a 12 K sum spectrum from three clusters, where the other two spectra were taken to be Gaussian. This is inconsistent with basic physics. Lorentzian line shapes are always associated with relaxational lifetimes, which implies that taking a sample from 40 to 12 K would result in a much sharper Lorentzian. The spectral deconvolution is therefore meaningless. In a subsequent study on bovine Complex I, the Lorentzian fit to N1b was reverted to a Gaussian without further comments [34].

**Figure 12:**
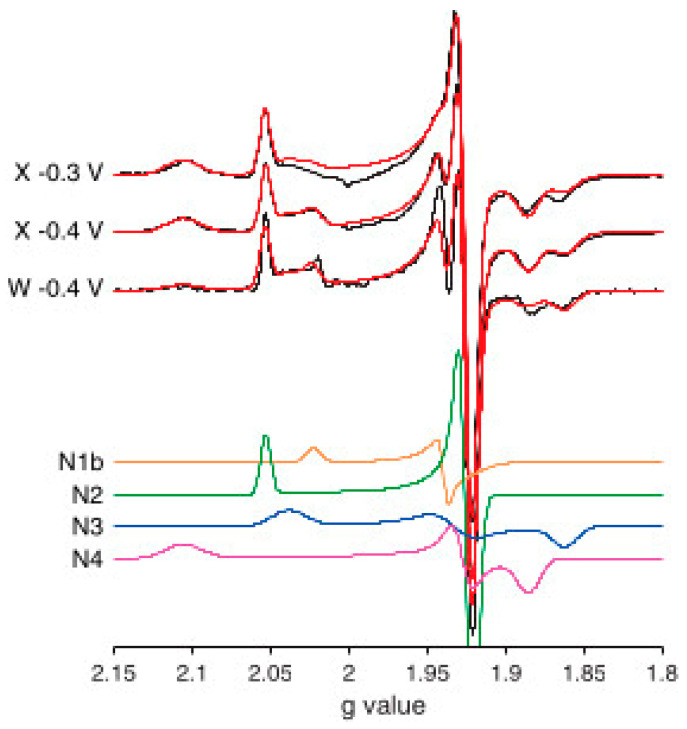
EPR spectra and simulations of NADH-reduced Complex I of bovine-heart tissue from Roessler’s and Hirst’s groups. Spectra were recorded in X-band (9.40 GHz) and W-band (93.9 GHz). Sum-spectra were constructed from single-component spectra generated under the assumption of Gaussian broadening symmetric in *B*-space and with the linewidth re-calculated in *g*-value units. Stoichiometries resulting from the analysis of 10 mM NADH-reduced enzyme (−0.4 V) were N1b:N2:N3:N4 = 0.28:1.00:1.03:0.81 when normalized on N2’s spin count. Reprinted from [20]; Copyright National Academy of Sciences.

In 2010, the combined groups of Maxie Roessler and Judy Hirst reperformed the analysis of bovine Complex I, but this time used *g*-strain to describe inhomogeneous broadening. Line shapes were Gaussian symmetrical on a *B*-scale and line widths were re-calculated in *g*-value units (that is, symmetry in *g*-space and linewidth non-colinearity was ignored). Their result is reproduced in Figure 12).

The percentage contributions of N1b, N2, N3, N4 at a potential of −0.4V (−0.3V) were 9 (3), 32 (34), 26 (23), 33 (40). In words: the spin count of N1b was clearly low and that of N3 was a bit on the low side. This is essentially the same result as that obtained 33 years earlier by Albracht [59]. No progress in theoretical description of broadening (my *g*-strain papers were passed over) means no progress in analytical results. In subsequent more recent work by these authors, the methodology of EPR analysis has not changed [60,65]. In my view, the inescapable conclusion is that analysis of *g*-strained complex spectra has come to a halt.

## 7. Complex-I: Differential Saturation of Other Clusters

**Figure 13:**
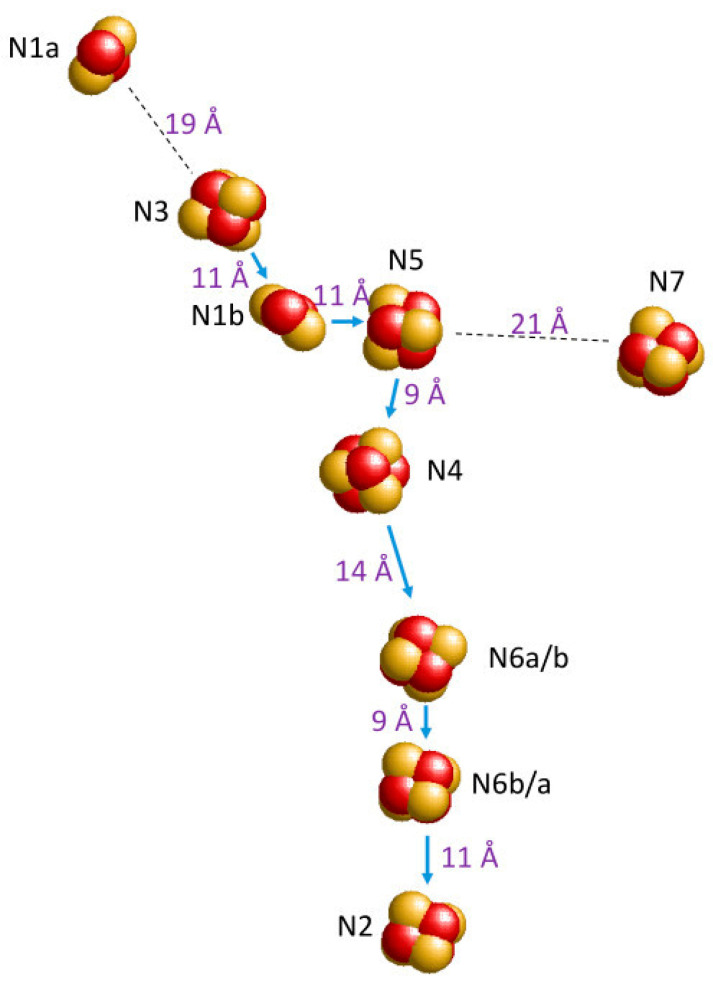
Nine iron–sulfur clusters in the crystal structure of the hydrophilic domain of *Thermus thermophilus* Complex I. Iron is coded in bright red; sulfur is in yellow. Putative electron-transfer route from N3 to N2 is indicated with blue arrows. Clusters N1a and N7 are not part of this pathway. N7 is not present in Complex 1 from many other species. Edge-to-edge distances (in violet) are indicated in ångström. Figure constructed from 2FUG.pdb [74,75].

Complex-spectrum deconvolution in the previous section was limited to four components, which we labeled N1b, N2, N3, and N4. However, protein-structure resolution by means of crystallography and electron microscopy has revealed that Complex I, depending on species, contains 7–9 iron–sulfur clusters [74,75,76,77,78,79,80,81,82,83,84,85]. For example (Figure 13), the crystal structure at 3.3 Å resolution of the hydrophilic domain of *Thermus thermophilus* Complex I (2FUG.pdb) discloses the 3D arrangement of nine clusters [74,75].

Later comparison with complex-I’s from other species indicated that occurrence of N7 is limited to the *T. thermophilus* and *E. coli* systems, but the other eight clusters are common to all. What, then, is the EPR of clusters N1a, N5, N6a, N6b?

**Figure 14:**
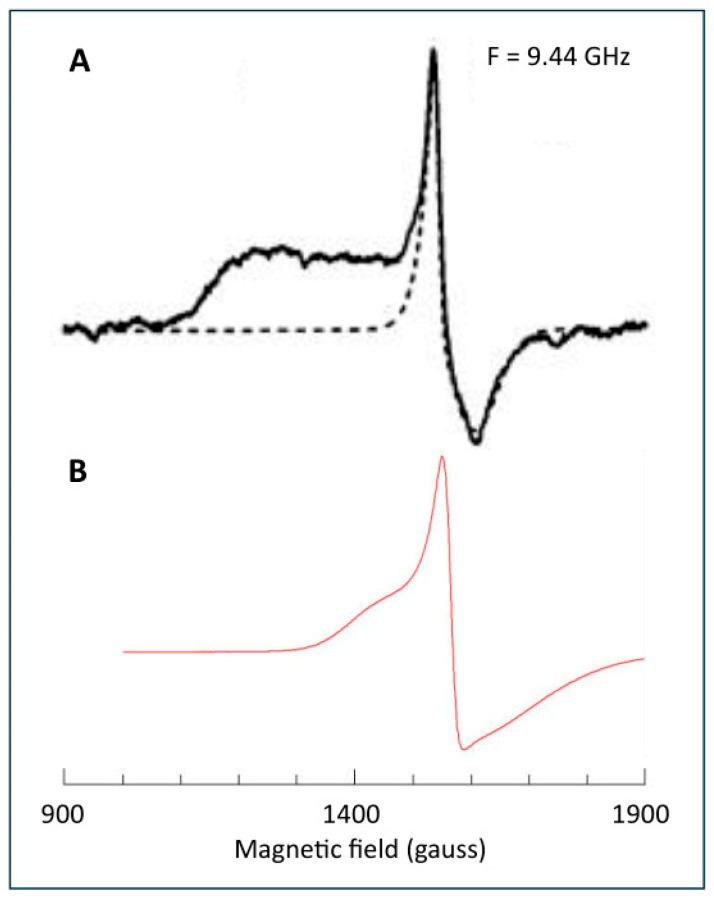
Putative *S* =3/2 signal ascribed to center N5 by Ohnishi’s group. Trace A is from subunit Nqo3 from Complex I of *Paracoccus denitrificans* measured under extreme conditions of low temperature (5 K) and high microwave power (100 mW). The dotted line is a simulation of an Fe(III) contaminant as an effective *S* = 1/2 system with axial *g*-tensor *g*_z_ = 4.362 and *g*_⊥_ = 4.236, 4.236, which is an unphysical solution to an Fe(III) d^5^ system with quenched orbital angular momentum. Trace B is the spectrum (7 K; 200 mW) of an arbitrarily chosen rhombic high-spin Fe(III) system (rubredoxin from *Megasphaera elsdenii*) to illustrate the broad wings that are usually exhibited by this type of spectra. Trace A was reprinted from [87] with permission (CC-BY).

In a mini-review in 1993, Ohnishi cursorily announced that ‘EPR signals of some clusters, such as cluster N5, can be substoichiometric to the complex or undetectable due to the dominant *S* = 3/2 ground state of their spin’ [86]. Five years later, in another mini-review, she elaborated that ‘while cluster N5 concentration is only about 0.25 to one FMN… cluster N5 may have an equivalent spin concentration with that of FMN, but 75% of the N5 spins are EPR non-detectable because they may be in the *S* = 3/2 ground state’ [70]. Ten years later (2003), the expected *S* = 3/2 EPR was finally detected in subunit Nqo3 of Complex I from *Paracoccus denitrificans* (see Figure 14A) [87].

Interference was suggested with a *g* = 4.3 signal from rhombic Fe(III) ions, and in order to obtain a clean *S* = 3/2 spectrum, the contaminating signal was simulated with *g*_z,y,x_ = 4.363, 4.236, 4.236, and linewidths *L*_z,y,x_ = 12, 105, 75 gauss, for subtraction from the original data. Once more, this is inconsistent with basic physics. High-spin Fe(III) ions have a 3*d*^5^ half-filled outer shell and thus have a *g*-value very close to the free-electron value g_e_ = 2.002 due to quenching of orbital angular momentum. This means that the effective *g*-values can be accurately predicted with rhombograms [88,89], to which the simulated axial effective *g*-tensor in Figure 14 is not a solution. Furthermore, these spectra are broadened by a distribution in rhombicity [90], resulting in a relatively sharp signal flanked by two broad wings (compare the spectrum of a ferric rubredoxin that I added to Figure 14). I suggest that the Ohnishi group has erroneously construed part of a contaminating 4.3 signal (namely, the low-field wing) as stemming from an *S* = 3/2 signal; the signal has nothing to do with cluster N5. Later, Nakamaru-Ogiso et al. [91], as well as Yakovlev et al. [33], specifically mentioned their inability to detect any *S* = 3/2 EPR in Complex I subunit NuoG from *E. coli*, and no new reports on *S* = 3/2 in any Complex I or derivative have appeared over the last two decades.

**Figure 15:**
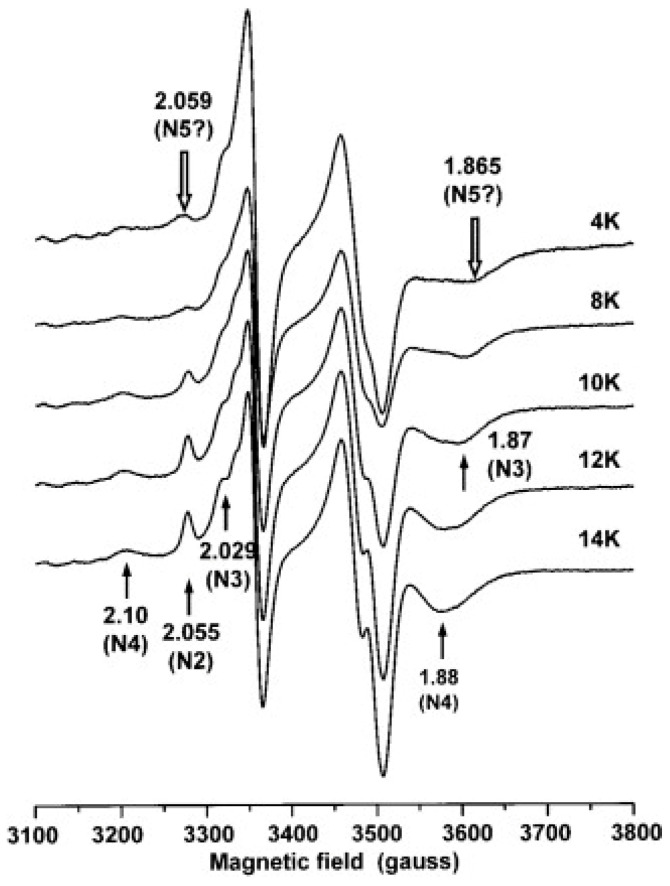
EPR spectra from Complex I-enriched *P. denitrificans* membranes as a function of temperature. In these data, the Ohnishi group thought to detect signals from cluster N5 at low temperature (4 K) and high microwave power (10 mW). Figure reprinted from ref. [87] with permission (CC-BY).

There is a second part to the N5 story. In her 1998 mini-review, Ohnishi stated that the EPR of N5 in bovine-heart Complex I is actually a mixture of *S* = 3/2 (75%) and *S* = 1/2 (25%), with the latter having *g*-values of 2.07, 1.93, and 1.90 and detectability limited to *T* < 7 K [70]. Actual data were published in 2003 (Figure 15) for *P. denitrificans* membranes [87].

The *g*-values had now changed to *g*_z_ = 2.059, *g*_y_ unidentified, and *g*_x_ = 1.865, and the two peaks were slightly shifted with respect to peaks, detected at higher temperatures, from N2 and N4 (N3 in Ohnishi’s nomenclature). The EPR conditions to detect N5, namely, *T* = 4 K and microwave power 10 mW, are unusual, to say the least, for the detection of an *S* = 1/2 iron–sulfur cluster, because they are likely to be highly saturating. The experiment, in fact, is a clear example of the manifestation/consequence 4 of *g*-strain (see Section 4), and thus also non-observance of proper analysis of *g*-strained spectra: a deformation of spectral features by differential saturation resulting in an *apparent* shift in peak position. All in all, the EPR of N5, in my view, is an illusion based on methodological error. The *S* = 1/2 spectral deformation itself is, of course, real and has been repeatedly reproduced by several groups [33,64,92,93,94,95,96,97].

The EPR of the clusters N6a and N6b has never been observed until now except for the Complex I and derivatives from *E. coli*. In 2007, Belevich et al. were the first to report a signal from *E. coli* Complex I reduced with NADH with *g*-values of 1.889, 1.905, and 2.087 (from a simulation in which *g*_y_ and *g*_x_ were not resolved), measured at 10 K and 10 mW power, and they tentatively assigned it to be cluster N6b [98]. They did not discuss, however, the fact that N4 in *E. coli* Complex I has very similar *g*-values 1.89, 1.93, and 2.09 [69], and that discrimination based on power saturation runs the obvious risk of violating the limitations set by *g*-strain theory.

**Figure 16:**
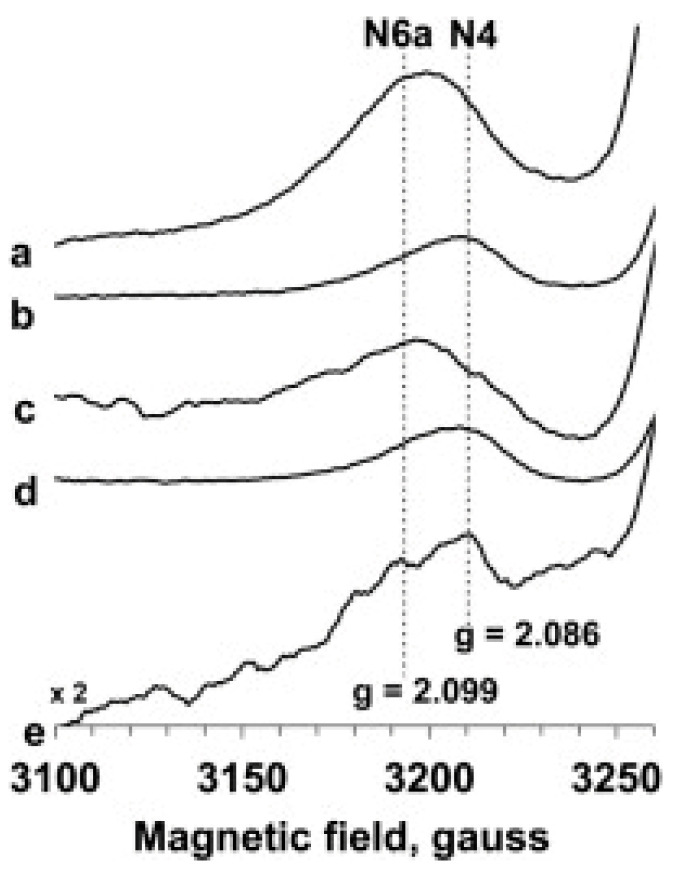
EPR spectra of *E. coli* Complex 1 at 10 K and as a function of microwave power. For traces a, c the power was 204 mW; for traces b, d the power was 1.2 mW. The reductant was 20 mM dithionite (a, b) or 6 mM NADH (c, d). Trace e is a difference spectrum (dithionite-reduced minus NADH-reduced) at 81 mW. In these data, the group of Nakamaru-Ogiso believed they had detected a signal from cluster N6a. Figure reprinted from [99] with permission (CC-BY).

Ohnishi then cited this result in a review as 1.89, 1.94, and 2.09 (note the misquote in the *g*_y_ value) and left out the label ‘tentatively’, thus giving it the status of an undisputed assignment [63]. In 2012, the Ohnishi group re-assigned the *g*_z_ = 2.091 (the other two *g*-values were obscured) from *E. coli* Complex I, but now reduced with dithionite plus viologen mediators, to cluster N6a based on site-directed-mutagenesis studies, and assigned a different set *g*_z_ = 2.05 and *g*_x,y_ ≈ 1.90 to cluster N6b [96] (note that N2 also has *g*_z_ = 2.05 [69]). These authors argue that N4 should not contribute significantly under the employed conditions of low temperature and high power, a statement that once more would seem to violate *g*-strain limitations. Perhaps an even clearer illustration of not observing the boundary conditions set by *g*-strain theory is found in the subsequent (2013) work by Narayanan et al. who shift the peak position of an asymmetric absorption feature with high power and then assign the original peak and the shifted peak, respectively, to cluster N4 and cluster N6a (Figure 16) [99].

The bottom line seems to be that putative signals from *E. coli* Complex I only, recorded at high microwave power and overlapping with established signals from N2 (*g* = 2.05) and N4 (*g* = 2.09), have been assigned to N6a and N6b under conditions that are strongly dissuaded by *g*-strain theory. My conclusion: the reported EPR signals from clusters N6a and N6b are artifacts of differential saturation.

In 1975, Ohnishi reported on the EPR of a low-potential cluster N1a [58]. A [2Fe-2S] cluster signal in Complex I in pigeon-heart mitochondria consisted of two very similar signals from two different centers with *g*_x,y_ =1.94; *g*_z_ = 2.03 (N1a) and *g* = 1.91; 1.94; 2.03 (N1b), which could be disentangled based on their reduction potential at pH 7.2, −396 mV (N1a) and −231 mV (N1b). N1b was thus reducible with NADH, but N1a could only be reduced with dithionite plus mediators. In a subsequent paper in 1981, she reported E_m_’s for bovine-heart Complex I at pH 8.0 as <−500 mV (N1a) and −335 mV (N1b). Other groups looked for N1a EPR in purified Complex I, but were unable to detect any [59,62,94], not even with the very strong reductant EDTA/deazaflavin/light [62]. Later (1993), the Ohnishi group reported in a review paper *g*-values and E_m_’s also for several bacterial complexes, partially as ‘unpublished data’ [92]. The fact of the matter is that no group other than Ohnishi’s has thus far reported on N1a EPR, with two exceptions. Firstly, Hirst’s group did direct electrochemistry on a 24 kDa subunit (NQO2) purified from Complex I from four different sources, and they ascribed the EPR to cluster N1a, being careful to point out that ‘It is likely that, since the cluster is exposed on the tip of the protein fold, the spectroscopic characteristics are modified upon removal of the subunit from Complex-I interior’ [100]. Secondly, where the Ohnishi group originally reported on EPR of N1a, N1b, and N1c centers in *E. coli* Complex I [69,92], Uhlmann and Friedrich later made a case that ‘N1c’ should actually be assigned to N1a, which, moreover, was simply reducible with NADH [71]. The question remains as to what the data for N1a, reported in [69,92] would refer to. Taken together, I would venture that the occurrence of N1a EPR in intact Complex I (with the exception of the *E. coli* complex) is still awaiting confirmation by groups other than Ohnishi’s. *g*-Strain, and quantification based on *g*-strain broadening, has thus far never been considered for N1a signals.

Finally, the primary sequences of *E. coli* and *T. thermophilus* Complex I bear a four-cysteine binding motif labeled N7, which is absent in the bovine-heart enzyme. When the joined groups of Ohnishi and Yagi (2002) purified subunit Nqo3 from *T. thermophilus* carrying this motif and reconstructed with added iron and sulfide, in addition to the [2Fe-2S] N1b signal, a mix of [4Fe-4S] cluster signals was observed and assigned to cluster N7 [101]. A somewhat similar [4Fe-4S] signal from N7 was later found by the Hirst group in subunit NuoG from *E. coli* Complex I and was simulated using a colinear linewidth tensor and a line shape, linear in *B*-space [33]. The EPR of the N7 cluster has yet to be identified in any intact Complex I that contains the binding motif.

## 8. Conclusions

Forty years ago, after we completed the development of the statistical theory of *g*-strain, I picked up the case of Complex I as an example to explore the scope of the theory’s applicability [11]. The outcome of the analysis was simple and straightforward: the EPR of NADH-reduced complex from bovine-heart mitochondria is near-perfectly reproduced as a stoichiometric sum of four *g*-strained iron–sulfur signals N1a-N2-N3-N4. No other iron–sulfur signals (now, e.g., known as N1a, N5, N6a, N6b) contributed to the spectrum. Regrettably, our analysis was universally overlooked by the communities of enzymologists and EPR spectroscopists, and thus an unrelated research effort emerged, and continued up to today, in which attempts at stoichiometry determinations were based on erroneous theories of inhomogeneous broadening, and in which attempts at making EPR-silent clusters detectable were based on the erroneous assumption that ever-lower detection temperatures combined with ever-higher microwave power levels would create situations in which differential saturation would be a legitimate methodology for disentangling complex overlapping spectral patterns.

In the exemplary case of Complex I, significant progress has been made in our knowledge of the structure and mechanism of action of the enzyme; however, basic analysis of its EPR spectroscopy has been at an essential standstill for four decades. It is my sincere wish that the here-presented updated recap of *g*-strain theory will incite the community to a timely key correction in methodology, such that I do not have to bite my nails for another forty years.

## Figures and Tables

**Table 1:** Sets of *g*-values for the iron–sulfur centers in NADH-reduced Complex I reported from different analyses. The three tabulated sets are based on different broadening mechanisms, namely, from Albracht et al. (1977) based on unresolved hyperfine interactions, from Hagen et al. (1985) based on non-colinear *g*-strain symmetrical in *g*-space, and from Clifford et al. (2025) based on colinear *g*-strain symmetrical in *B*-space. Data are from [11,59,60].

Center (^a^)	*g*_x_	*g*_y_	*g*_z_	Author
N1b	1.9278–1.9381		2.0213	(Albracht et al. [59])
	1.925	1.937	2.019	(Hagen et al. [11])
	1.923	1.941	2.022	(Clifford et al. [60])
N2	1.925	1.925	2.0538	[59]
	1.925	1.925	2.053	[11]
	1.922	1.928	2.055	[60]
N3	1.884	1.938	2.103	[59]
	1.887	1.941	2.104	[11]
	1.883	1.929	2.102	[60]
N4	1.863	1.9263	2.037	[59]
	1.862	1.921	2.034	[11]
	1.864	1.928	2.039	[60]

(^a^) In ref. [60], the labeling of N3 and N4 is swapped.

## Data Availability

No new data were generated for this review.

## References

[1] Ponti A. (1999). Simulation of magnetic resonance static powder lineshapes: A quantitative assessment of spherical codes. J. Magn. Reson..

[2] Hagen W.R. (2021). Very low-frequency broadband electron paramagnetic resonance spectroscopy of metalloproteins. J. Phys. Chem. A.

[3] Hagen W.R. (1976). Een Onderzoek Naar de Electron Paramagnetische Resonantie Eigenschappen van Cytochroom c Oxidase. Master’s Thesis.

[4] Johnston T.S., Hecht H.G. (1965). An automated fitting procedure for the determination of anisotropic *g*-tensors from EPR studies of powder samples. J. Mol. Spectrosc..

[5] Venable J.H., Ehrenberg A., Malmström B.G., Vänngård T. (1967). Electron paramagnetic resonance spectroscopy of protein single crystals: II Computational methods. Magnetic Resonance in Biological Systems, Proceedings of the Second International Conference Held at the Wenner-Gren Center Stockholm, Stockholm, Sweden, 9–15 June 1966.

[6] Aasa R., Vänngård T. (1975). EPR signal intensity and powder shapes: A reexamination. J. Magn. Reson..

[7] Aasa R., Albracht S.P.J., Falk K.-E., Lanne B., Vänngård T. (1976). EPR signals from cytochrome c oxidase. Biochim. Biophys. Acta.

[8] Hagen W.R. (1981). Dislocation strain broadening as a source of anisotropic linewidth and asymmetrical lineshape in the electron paramagnetic resonance spectrum of metalloproteins and related systems. J. Magn. Reson..

[9] Hagen W.R., Hoff A.J. (1989). g-Strain: Inhomogeneous broadening in metalloprotein EPR. Advanced EPR.

[10] Hagen W.R., Albracht S.P.J. (1982). Analysis of strain-induced EPR-line shapes and anisotropic spin-lattice relaxation in a [2Fe-2S] ferredoxin. Biochim. Biophys. Acta.

[11] Hagen W.R., Hearshen D.O., Harding L.J., Dunham W.R. (1985). Quantitative numerical analysis of g strain in the EPR of distributed systems and its importance for multicenter metalloproteins. J. Magn. Reson..

[12] Pilbrow J.R. (1984). Lineshapes in frequency-swept and field-swept EPR for spin 1/2. J. Magn. Reson..

[13] Hagen W.R. (1982). Electron Paramagnetic Resonance of Metalloproteins; with Emphasis on Components of the Respiratory Chain. Ph.D. Thesis.

[14] Pake G.E., Estle T.L. (1973). The Physical Principles of Electron Paramagnetic Resonance.

[15] Hagen W.R., Hearshen D.O., Sands R.H., Dunham W.R. (1985). A statistical theory for powder EPR in distributed systems. J. Magn. Reson..

[16] Hearshen D.O., Hagen W.R., Sands R.H., Grande H.J., Crespi H.L., Gunsalus I.C., Dunham W.R. (1986). An analysis of g strain in the EPR of two [2Fe-2S] ferredoxins. Evidence for a protein rigidity model. J. Magn. Reson..

[17] Wiertz F.G.M., Richter O.-M.H., Cheropanov A.V., MacMillan F., Ludwig B., de Vries S. (2004). An oxo-ferryl tryptophan radical catalytic intermediate in cytochrome *c* and quinol oxidases trapped by microsecond freeze-hyperquenching (MHQ). FEBS Lett..

[18] Srour B., Strampraad M.J.F., Hagen W.R., Hagedoorn P.-L. (2018). Refolding kinetics of cytochrome *c* studied with microsecond timescale continuous-flow UV-vis spectroscopy and rapid freeze-quench EPR. J. Inorg. Biochem..

[19] Vinogradov A.D., Sled V.D., Burbaev D.S., Grivennikova V.G., Moroz I.A., Ohnishi T. (1995). Energy-dependent complex I-associated ubisemiquinones in submitochondrial particles. FEBS Lett..

[20] Roessler M.M., King M.S., Robinson A.J., Armstrong F.A., Harmer J., Hirst J. (2010). Direct assignment of EPR spectra to structurally defined iron-sulfur clusters in complex I by double electron-electron resonance. Proc. Natl. Acad. Sci. USA.

[21] Narayanan M., Leung S.A., Inaba Y., Elguindy M.M., Nakamaru-Ogiso E. (2015). Semiquinone intermediates are involved in the energy coupling mechanism of *E coli* complex I. Biochim. Biophys. Acta.

[22] Beinert H., Orme-Johnson W.H., Ehrenberg A., Malmström B.G., Vänngård T. (1967). Electron spin relaxation as a probe for active centers of paramagnetic enzyme species. Magnetic Resonance in Biological Systems, Proceedings of the Second International Conference Held at the Wenner-Gren Center Stockholm, Stockholm, Sweden, 9–15 June 1966.

[23] Rupp H., Rao K.K., Hall D.O., Cammack R. (1978). Electron spin relaxation of iron-sulphur proteins studied by microwave power saturation. Biochim. Biophys. Acta.

[24] Portis A.M. (1953). Electronic structure of *F* centers: Saturation of the electron spin. Phys. Rev..

[25] Albracht S.P.J., Leeuwerik F.J., van Swol B. (1979). The stoichiometry of the iorn-sulphur clusters 1a, 1b and 2 of NADH:Q oxidoreductase as present in beef-heart submitochondrial particles. FEBS Lett..

[26] Petrovich R.M., Ruzicka F.J., Reed G.H., Frey P.A. (1992). Characterization of iron-sulfur clusters in lysine 2,3-aminomutase by electron paramagnetic resonance spectroscopy. Biochemistry.

[27] Kjaer B., Jung Y.-S., Yu L., Golbeck J.H., Scheller H.V. (1994). Iron-sulfur centers in the photosynthetic reaction center complex from *Chloronium vibrioforme*. Differences from and similarities to the iron-sulfur centers in photosystem I. Photosys. Res..

[28] Broderick J.B., Duderstadt R.E., Fernandez D.C., Wojtuszewski K., Henshaw T.F., Johnson M.K. (1997). Pyruvate formate-lyase activating enzyme is an iron-sulfur protein. J. Am. Chem. Soc..

[29] Vassiliev I.R., Antonkine M.L., Golbeck J.H. (2001). Iron-sulfur clusters in type I reaction centers. Biochim. Biophys. Acta.

[30] Madadi-Kahkesk S., Duin E.C., Heim S., Albracht S.P.J., Johnson M.K., Hedderich R. (2001). A paramagnetic species with unique EPR characteristics in the active site of heterodisulfide reductase from methanogenic archaea. Eur. J. Biochem..

[31] Duin E.C., Bauer C., Jaun B., Hedderich R. (2003). Coenzyme M binds to a [4Fe-4S] cluster in the active site of heterodisulfide reductase as deduced from EPR studies with the [^33^S]coenzyme M-treated enzyme. FEBS Lett..

[32] Agarwalla S., Stroud R.M., Gaffney B.J. (2004). Redox reactions of the iron-sulfur cluster in a ribosomal RNA methyltransferase, RumA. J. Biol. Chem..

[33] Yakovlev G., Reda T., Hirst J. (2007). Reevaluating the relationship between EPR spectra and enzyme structure for the iron-sulfur clusters in NADH:quinone oxidoreductase. Proc. Natl. Acad. Sci. USA.

[34] Reda T., Barker C.D., Hirst J. (2008). Reduction of the iron-sulfur clusters in mitochondrial NADH:ubiquinone oxidoreductase (Complex I) by Eu^II^-DTPA, a very low potential reductant. Biochemistry.

[35] Kondo T., Nomata J., Fujita Y., Itoh S. (2011). EPR study of 1Asp-3Cys ligated 4Fe-4S iron-sulfur cluster in NB-protein (BchN-BchB) of a dark-operative protochlorophyllide reductase complex. FEBS Lett..

[36] Stich T.A., Dos Santos P.C. (2021). Characterization of paramagnetic iron-sulfur clusters using electron paramagnetic resonance spectroscopy. Fe-S Proteins.

[37] Priem A.H., Klaassen A.A.K., Reijerse E.J., Meyer T.E., Luchinat C., Capozzi F., Dunham W.R., Hagen W.R. (2005). EPR analysis of multiple forms of [4Fe-4S]^3+^ clusters in HiPIPs. J. Biol. Inorg. Chem..

[38] Blaszczyk A.J., Silakov A., Zhang B., Mailcco S.J., Lanz N.D., Kelly W.L., Elliott S.J., Krebs C., Booker S.J. (2016). Spectroscopic and electrochemical characterization of the iron-sulfur and cobalamin cofactors of TsrM, and unusual radical s-adenosylmethionine methylase. J. Am. Chem. Soc..

[39] Barr I., Stich T.A., Gizzi A.S., Grove T.L., Bonanno J.B., Latham J.A., Chung T., Wilmot C.M., Britt C.D., Almo S.C. (2018). X-ray and EPR characterization of the auxiliary Fe-S clusters in the radical SAM enzyme PqqE. Biochemistry.

[40] Heghmanns M., Günzel A., Brandis D., Kutin Y., Engelbrecht V., Wikler M., Happe T., Kasanmascheff M. (2021). Fine-tuning of FeS proteins monitored via pulsed EPR redox potentiometry at Q-band. Biophys. Rep..

[41] Laurikenas A., Sakalauskas D., Marsalka A., Raudonis R., Antuzevics A., Balevidus V., Zarkov A., Karelva A. (2021). Investigation of lanthanum substitution effects in yttrium aluminium garnet: Importance of solid state NMR and EPR methods. J. Sol-Gel Sci. Technol..

[42] Paulin J.V., Batagin-Neto A., Naydenov B., Lips K., Graeff C.F.O. (2021). High-field/high frequency EPR spectroscopy on synthetic melanin: On the origin of carbon-centered radicals. Mater. Adv..

[43] Dubroca T., Wang X., Mentink-Vigier F., Trociewitz B., Starck M., Parker D., Sherwin M.S., Hill S., Krzystek J. (2023). Terahertz EPR spectroscopy using a 36-tesla high-homogeneity series-connected hybrid magnet. J. Magn. Reson..

[44] Tran V.A., Teucher M., Galazzo L., Sharma B., Pongratz T., Kast S.M., Marx D., Bordignon E., Schnegg A., Neese F. (2023). Dissecting the molecular origin of g-tensor heterogeneity and strain in nitroxide radicals in water: Electron paramagnetic resonance experiment versus theory. J. Phys. Chem. A.

[45] Maio N., Raza M.K., Li Y., Zhang D.-L., Bollinger J.M., Krebs C., Rouault T.A. (2023). An iron-sulfur cluster in the zink-binding domain of the SARS-CoV-2 helicase modulates its RNA-binding and -unwinding activities. Proc. Natl. Acad. Sci. USA.

[46] Santanni F., Little E., Lockyer S.J., Whitehead G.F.S., McInnes E.J.L., Timco G.A., Bowen A.M., Sessoli R., Winpenny R.E.P. (2024). Weak exchange interactions in multispin systems: EPR studies of metalloporphyrins decorated with {Cr_7_Ni} rings. Inorg. Chem..

[47] Kultaeva A., Biktagirov T., Sperlich A., Dörfinger P., Calvo M.E., Otal E., Dyakonov V. (2025). Photoinduced spin centers in photocatalytic metal-organic framework UiO-66. Adv. Funct. Mater..

[48] Stoll S., Schweiger A. (2006). EasySpin, a comprehensive software package for spectral simulation and analysis in EPR. J. Magn. Reson..

[49] Stoll S. (2015). CW-EPR spectral simulations: Solid state. Methods Enzymol..

[50] Beinert H., Sands R.H. (1960). Studies on succinic and DPNH dehydrogenase preparations by paramagnetic resonance (EPR) spectroscopy. Biochem. Biophys. Res. Commun..

[51] Ohnishi T., Asakura T., Wohlrab H., Yonetani T., Chance B. (1970). Electron paramagnetic resonance studies on iron-sulfur proteins of submitochondrial particles from *Candida utilis* cells. J. Biol. Chem..

[52] Albracht S.P.J., Slater E.C. (1971). EPR studies at 20^0^ K on the mitochondrial respiratory chain. Biochim. Biophys. Acta.

[53] Orme-Johnson N.R., Orme-Jonson W.H., Hansen R.E., Beinert H. (1971). EPR detectable electron acceptors in submitochondrial particles from beef heart with special reference to the iron-sulfur components of DPNH-ubiquinone reductase. Biochem. Biophys. Res. Commun..

[54] Ohnishi T., Asakura T., Yonetani T., Chance B. (1971). Electron paramagnetic resonance studies at temperatures below 77 °K on iron-sulfur proteins of yeast and bovine heart submitochondrial particles. J. Biol. Chem..

[55] Ohnishi T., Wilson D.F., Asakura T., Chance B. (1974). Studies on iron-sulfur proteins in the site I region of the respiratory chain in pigeon heart mitochondria and submitochondrial particles. Biochem. Biophys. Res. Commun..

[56] Albracht S.P.J. (1974). Some new paramagnetic centers in submitochondrial particles detectable by EPR spectroscopy. Biochim. Biophys. Acta.

[57] Orme-Johnson N.R., Hansen R.E., Beinert H. (1974). Electron paramagnetic resonance-detectable electron acceptors in beef heart mitochondria. Reduced diphosphopyridine nucleotide ubiquinone reductase segment of the electron transfer system. J. Biol. Chem..

[58] Ohnishi T. (1975). Thermodynamic and EPR characterization of iron-sulfur centers in the NADH-ubiquinone segment of the mitochondrial respiratory chain in pigeon heart. Biochim. Biophys. Acta.

[59] Albracht S.P.J., Dooijewaard G., Leeuwerik F.J., van Swol B. (1977). EPR signals of NADH:Q oxidoreductase. Shape and intensity. Biochim. Biophys. Acta.

[60] Clifford E.R., Wright J.J., Collauto A., Hirst J., Roessler M.M. (2025). Characterization of a semiquinone radical bound in the active site of respiratory complex I by hyperfine spectroscopy. ChemRxiv.

[61] Hearshen D.O., Dunham W.R., Albracht S.P.J., Ohnishi T., Beinert H. (1981). EPR spectral simulation on cluster N-1b in NADH-ubiquinone oxidoreductase of bovine heart mitochondria. FEBS Lett..

[62] Kowal A.T., Morningstar J.E., Johnson M.K., Ramsay R.R., Singer T.P. (1986). Spectroscopic characterization of the number and type of iron-sulfur clusters in NADH:ubiquinone oxidoreductase. J. Biol. Chem..

[63] Ohnishi T., Nakamaru-Ogiso E. (2008). Were there any “misassignments” among iron-sulfur clusters N4, N5 and N6b in NADH-quinone oxidoreductase (complex I)?. Biochim. Biophys. Acta.

[64] Ohnishi T., Ohnishi S.T., Salerno J.C. (2018). Five decades of research on mitochondrial NADH-quinone oxidoreductase (complex I). Biol. Chem..

[65] Jarman O.D., Biner O., Wright J.J., Hirst J. (2021). *Paracoccus denitrificans*: A genetically tractable model system for studying respiratory complex I. Sci. Rep..

[66] Bridges H.R., Bill E., Hirst J. (2012). Mössbauer spectroscopy of respiratory complex I: The iron-sulfur cluster ensemble in the NADH-reduced enzyme is partially oxidized. Biochemistry.

[67] van Belsen R., Mariette A., Albracht S.P.J. (1992). On the stoichiometry of the iron-sulphur clusters in mitochondrial NADH: Ubiquinone oxidoreductase. Eur. J. Biochem..

[68] Albracht S.P.J., Mariette A. (1997). Bovine -heart NADH:ubiquinone oxidoreductase is a monomer with 8 Fe-S clusters and 2 FMN groups. Biochim. Biophys. Acta.

[69] Leif H., Sled V.D., Ohnishi T., Weiss H., Friedrich T. (1995). Isolation and characterization of the proton-translocating NADH: Ubiquinone oxidoreductase from *Escherichia coli*. Eur. J Biochem..

[70] Ohnishi T. (1998). Iron-sulfur clusters/semiquinones in Complex I. Biochim. Biophys. Acta.

[71] Uhlmann M., Friedrich T. (2005). EPR signals assigned to Fe/S cluster N1c of the *Escherichia coli* NADH:ubiquinone oxidoreductase (complex I) derive from cluster N1a. Biochemistry.

[72] Wang D.-C., Meinhardt S., Sackmann U., Weiss H., Ohnishi T. (1991). The iron-sulfur clusters in two related forms of mitochondrial NADH:ubiquinone oxidoreductase made by *Neurospora crassa*. Eur. J. Biochem..

[73] Lin T.-I., Sled V.D., Ohnishi T., Brennicke A., Grohmann L. (1995). Analysis of the iron-sulfur clusters within comple I (NADH:ubiquinone oxidoreductase) isolated from potato tuber mitochondria. Eur. J. Biochem..

[74] Hinchliffe P., Sazanov L.A. (2005). Organization of iron-sulfur clusters in respiratory complex I. Science.

[75] Sazanov L.A., Hinchliffe P. (2006). Structure of the hydrophilic domain of respiratory complex I from *Thermus thermophilus*. Science.

[76] Berrisford J.M., Sazanov L.A. (2009). Structural basis for the mechanism of respiratory complex I. J. Biol. Chem..

[77] Efremov R.G., Baradaran R., Sazanov L.A. (2010). The architecture of respiratory complex I. Nature.

[78] Vinothkumar K.R., Zhu J., Hirst J. (2014). Archirecture of mammalian respiratory complex I. Nature.

[79] Zhu J., Vinothkumar K.R., Hirst J. (2016). Structure of mammalian respiratory complex I. Nature.

[80] Feidorczuk K., Letts J.A., Degliesposti G., Kaszuba K., Skehel M., Sazanov L.A. (2016). Atomic structure of the entire mammalian mitochondrial complex I. Nature.

[81] Letts J.A., Fiedorczuk K., Degliesposti G., Skehel M., Sazanov L.A. (2019). Structures of respiratory supercomplex I+III_2_ reveal functional and conformational crosstalk. Mol. Cell.

[82] Soufari H., Parrot C., Kuhn L., Waltz F., Hashem Y. (2020). Specific features and assembly of the plant mitochondrial complex I revealed by cryo-EM. Nat. Commun..

[83] Parey K., Lasham J., Mills D.J., Djurabekova A., Haapanen O., Galemou Yoga E., Xie H., Kühlbrandt W., Sharma V., Vonck J. (2021). High-resolution structure and dynamics of mitochondrial complex I—Insights into the proton pumping mechanism. Sci. Adv..

[84] Gu J., Liu T., Guo R., Zhang L., Yang M. (2022). The coupling mechanism of mammalian mitochondrial complex I. Nat. Struct. Mol. Biol..

[85] Schimpf J., Oppermann S., Gerasimova T., Santos Seica A.F., Hellwig P., Grishkovskaya I., Wohlwend D., Haselbach D., Friedrich T. (2022). Structure of the peripheral arm of a minimalistic respiratory complex I. Structure.

[86] Ohnishi T. (1993). NADH-quinone oxidoreductase, the most complex complex. J. Bioenerg. Biomembr..

[87] Yano T., Sklar J., Nakamuru-Ogiso E., Takahashi Y., Yagi T., Ohnishi T. (2003). Characterization of cluster N5 as a fast-relaxing [4Fe-4S] cluster in the Nqo3 subunit of the proton-translocating NADH-ubiquinone oxidoreductase from *Paracoccus denitrificans*. J. Biol. Chem..

[88] Troup G.J., Hutton D.R. (1964). Paramagnetic resonance of Fe^3+^ in kyanite. Brit. J. Appl. Phys..

[89] Hagen W.R. (1992). EPR spectroscopy of iron-sulfur proteins. Adv. Inorg. Chem..

[90] Hagen W.R. (2007). Wide zero field interaction distributions in the high-spin EPR of metalloproteins. Mol. Phys..

[91] Nakamaru-Ogiso E., Yano T., Yagi T., Ohnishi T. (2005). Characterization of the iron-sulfur cluster N7 (N1c) in the subunit NuoG of the proton translocating NADH-quinone oxidoreductase from *Escherichia coli*. J. Biol. Chem..

[92] Sled V.D., Friedrich T., Leif H., Weiss H., Meinhardt S.W., Fukumori Y., Calhoun M.W., Gennis R.B., Ohnishi T. (1993). Bacterial NADH-quinone oxidoreductases: Iron-sulfur clusters and related problems. J. Bioenerg. Biomenbr..

[93] Djafarzadeh R., Kerscher S., Zwicker K., Radermacher M., Lindahl M., Schägger H., Brandt U. (2000). Biophysical and structural characterization of proton-translocating NADH-dehydrogenase (complexI) from the strictly aerobic yeast *Yarrowia lipolytica*. Biochim. Biophys. Acta.

[94] Kerscher S., Kashani-Poor N., Zwicker K., Zickermann V., Brandt U. (2001). Exploring the catalytic core of complex I by *Yarrowia lipolytica* yeast genetics. J. Bioenerg. Biomembr..

[95] Waletko A., Zwicker K., Abdrakhmanova A., Zickermann V., Brandt U. (2005). Histidine 129 in the 75-kDa subunit of mitochondrial complex I from *Yarrowia lipolytica* is not a ligand for [Fe_4_S_4_] cluster N5 but is required for catalytic activity. J. Biol. Chem..

[96] Sinha P.K., Nakamaru-Ogiso E., Torres-Bacete J., Sato M., Castro-Guerrero N., Ohnishi T., Matsuno-Yagi A., Yagi T. (2012). Electron transfer in subunit NuoI (TYKY) of *Escherichia coli* NADH:quinone oxidoreductase (NDH-1). J. Biol. Chem..

[97] Wright J.J., Salvadori E., Bridges H.R., Hirst J., Roessner M.M. (2016). Small-volume potentiometric titrations: EPR investigations of Fe-S cluster N2 in mitochondrial complex I. J. Inorg. Biochem..

[98] Belevich G., Euro L., Wikström M., Verkhovskaya M. (2007). Role of the conserved arginine 274 and histidine 224 and 228 residues in the NuoCD subunit of complex I from *Escherichia coli*. Biochemistry.

[99] Narayanan M., Gabrieli D., Leung S.A., Elguindy M.M., Glaser C.A., Saju N., Sinha S.C., Nakamaru-Ogiso E. (2013). Semiquinone and cluster N6 signals in his-tagged proton-translocating NADH:ubiquinone oxidoreductase (complex I) from *Escherichia coli*. J. Biol. Chem..

[100] Zu Y., Di Bernardo S., Yagi T., Hirst J. (2002). Redox properties of the [2Fe-2S] center in the 24 kDa (NQO2) subunit of NADH:ubiquinone oxidoreductase (complex I). Biochemistry.

[101] Nakamaru-Ogiso E., Yano T., Ohnishi T., Yagi T. (2002). Characterization of the iron-sulfur cluster coordinated by a cycteine cluster motif (CXXCXXXCX_27_C) in the Nqo3 subunit in the proton-translocating NADH:quinone oxidoreductase (NDH-1) of *Thermus thermophilus* HB-8. J. Biol. Chem..

